# Alcohol Oxidase–Imine Reductase Cascade for One-Pot Chiral Amine Synthesis

**DOI:** 10.1021/acscatal.5c06313

**Published:** 2026-01-14

**Authors:** Anya Miletic, Christopher J. Truby, Nicholas J. Turner, Rebecca E. Ruscoe

**Affiliations:** † Department of Chemistry, 5292 University of Manchester, Manchester Institute of Biotechnology, 131 Princess Street, Manchester M1 7DN, U.K.; ‡ School of Chemical and Physical Sciences & School of Allied Health Professions and Pharmacy, 4212 Keele University, Newcastle under Lyme, Keele, Staffordshire ST5 5BG, U.K.

**Keywords:** biocatalysis, reductive amination, conjugate reduction, biocatalytic cascades

## Abstract

We report a one-pot biocatalytic cascade for the conversion of allylic alcohols into enantioenriched secondary amines through a sequence involving oxidation followed by conjugate reduction–reductive amination (CR–RA). This transformation, catalyzed by a cholesterol oxidase (ShCO) and an ene-imine reductase (EneIRED), proceeds under mild conditions and accommodates a broad substrate scope. In several cases, the cascade outperforms the stepwise method and exhibits substrate-dependent selectivity, highlighting the advantages of tandem enzymatic systems for efficient and streamlined chiral amine synthesis.

## Graphical abstract

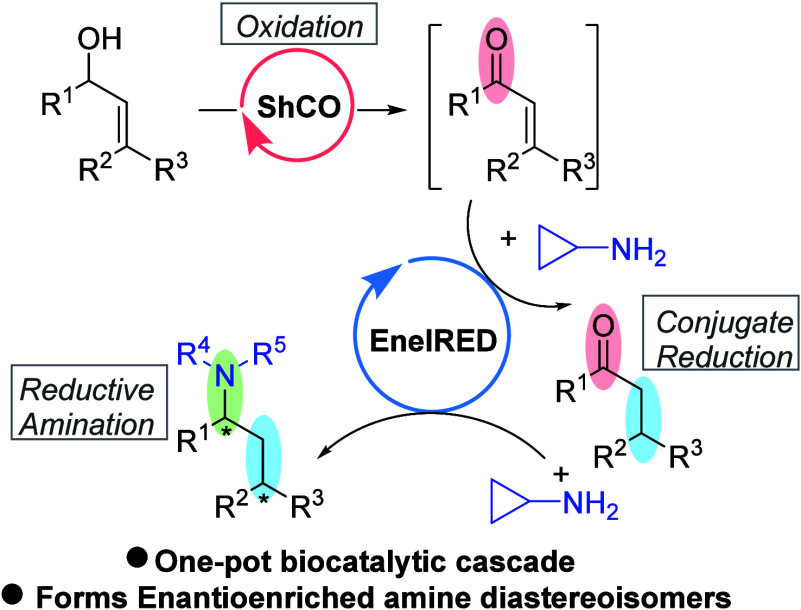

## 1 Introduction

Enantioenriched amine diastereoisomers are key structural motifs in numerous pharmaceutical and agrochemical compounds.^1^ Their synthesis often involves multiple steps, which can limit the overall efficiency. Reductive amination (RA) is a widely employed method for the introduction of chiral amines and is frequently integrated into multistep synthetic sequences targeting these valuable molecules.^2^

Biocatalysis has increasingly emerged as a powerful and versatile approach for enabling a wide range of chemical transformations.^3^ Given the importance of amine-containing compounds, considerable attention has been directed toward biocatalytic strategies for amine synthesis, including RA.^4,5^ Previously, we reported that screening a metagenomic panel of imine reductases (IREDs) resulted in the identification of enzymes capable of coupling aldehydes or ketones with amines.^6^ Subsequently, we showed that a subset of these IREDs were able to deliver hydride equivalents to both C=C and C=N functionalities, leading to their designation as EneIREDs.^7^ EneIREDs are multifunctional enzymes capable of sequential imine formation, alkene reduction, and imine reduction.

A key advantage of enzymes lies in their innate compatibility, allowing them to work in tandem in cascade reactions enabling multiple bond-forming steps to occur sequentially in a single vessel.^8–11^ Several groups have successfully combined IREDs and RedAms with other biocatalysts to develop such cascades.^12–18^ However, the ability of EneIREDs to exhibit their multifunctional reactivity within a tandem enzymatic system remains unexplored. Investigating this challenge not only offers deeper insight into the enzyme’s catalytic potential but also presents new opportunities for accessing enantioenriched amine diastereoisomers from a wider array of precursors.

Aldehydes and, to some extent, ketones are highly reactive intermediates, and their isolation from biocatalytic processes is often hindered by volatility and susceptibility to degradation. These factors also complicate their use as starting materials in subsequent transformations. To address these challenges, we designed a one-pot cascade process in which α,β-unsaturated aldehydes and ketones are generated *in situ* via biocatalytic oxidation, followed directly by an *in situ* conjugate reduction–reductive amination (CR-RA) sequence (Scheme 1). This strategy also leverages the advantages of using allylic alcohols, which are often commercially available or easily prepared from common precursors *via* established methods.^19,20^

**Scheme 1:**
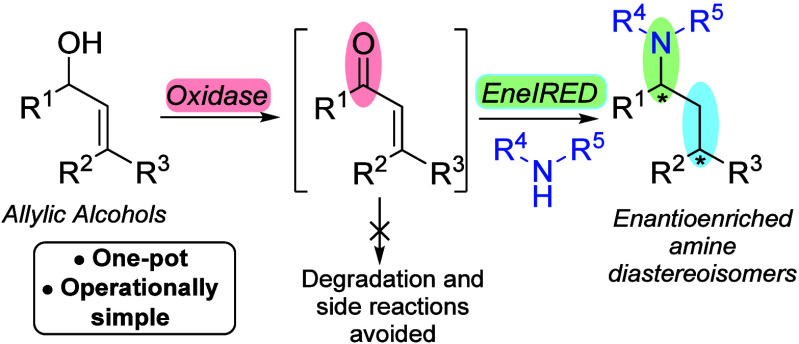
General Scheme for the Proposed Cascade

## 2 Results and Discussion

To achieve this, we began by exploring the oxidase step using 3-methylcyclohex-2-en-1-ol **1a** as a model substrate due to the fact that its enone derivative is known to have high activity in EneIRED-catalyzed CR–RA transformations.^7^ Additionally, an engineered cholesterol oxidase from *Streptomyces hygrospinosus* (ShCO_a_ – a two point mutant, see the Supporting Information) has been shown to efficiently oxidize secondary alcohols,^21^ including **1a**, making it a suitable starting point for this cascade.

Under the optimal conditions previously reported for the ShCO_a_ oxidase,^21^ the desired enone **2a** was obtained in excellent conversion (>99%) using ShCO_a_ (Table 1, Entry 1).To enable a one-pot cascade combining the oxidase step with an EneIRED, we switched to glycine buffer, as it consistently yielded high conversions in EneIRED-catalyzed reductive aminations (Table 1, Entry 2),^7^ which resulted in only a slight decrease in conversion (97%) at 10 mM **1a** compared to KPi buffer (Table 1, Entry 1). We then introduced 5% v/v DMSO, an essential cosolvent for the EneIRED-catalyzed cascade.^7^ Conversions remained high under these conditions, reaching 94% at 10 mM **1a** (Entry 3).

**Table 1:**
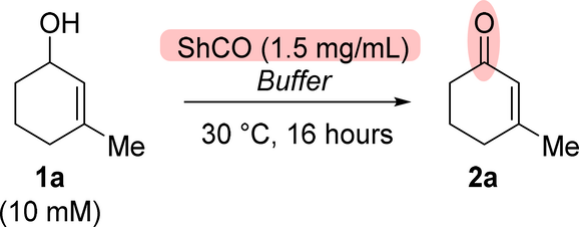
Exploring Conditions for the Oxidation of Allylic Alcohol **1a** Using ShCO^a^

condition	buffer	cosolvent	**2a (%)** ^b^
1	KPi		>99
2	Gly-OH		97
3	Gly-OH	DMSO	94

a 250 μL total volume with either KPi = 100 mM phosphate buffer pH 7.5 or Gly-OH = 100 mM glycine buffer pH 9.

b Conversions based on GC-FID.

We next explored the substrate scope of the oxidase for the formation of a range of enals and enones under the optimized conditions (Scheme 2). Encouragingly, a series of C3 substituted cyclohex-2-en-1-ols were tolerated (**1b**–**1g**), with the exceptions of substrates with cyclohexyl and phenyl groups at this position (**1d** and **1e**, respectively).

**Scheme 2:**
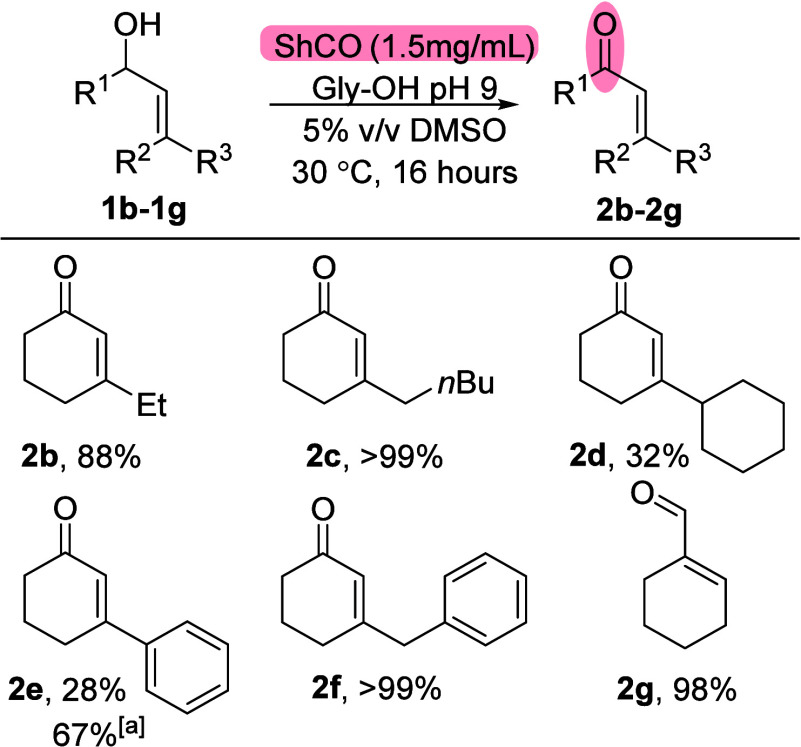
Scope of ShCO_a_ Oxidations^b^

a ShCO_b_ mutant used (see the Supporting Information).
b Conversions based on GCMS analysis.

The lower conversions observed for the cyclohexyl substituted allylic alcohol **1d** may be related to solubility, as precipitates were observed on the introduction of the starting material into the reaction medium. Although our previous work demonstrated that the oxidase enzyme tolerates various solvents,^21^ we did not pursue further optimization of this substrate by testing alternative solvents, which may have improved the conversion. The lower conversion for 3-phenyl substrate **1f** could be overcome by employing an alternative oxidase mutant (ShCO_b_),^21^ which showed a significant increase in conversion from 28% to 67% to the desired enone **2e** (see the Supporting Information). Interestingly, this limitation was not seen for the benzyl-substituted allylic alcohol **1f**, which formed the desired enone **2f** in conversions up to 99% without the need to utilize a different oxidase mutant.

We next investigated the formation of simple enal substrates. Allylic alcohol **1g** was efficiently converted to enal **2g** (98% conv.), but other low-molecular-weight allylic alcohols exhibited substantial degradation or competing side reactions under the reaction conditions or during the isolation of the product (see the Supporting Information).

Next, we explored combining ShCO_a_ and EneIRED in a one pot cascade approach (Figure 1). This required the addition of an amine coupling partner, NADP^+^ and a glucose dehydrogenase (GDH) recycling system. We focused first on C3-substituted cyclohex-ene-1-ols (**1a**–**1f**), given the preference of EneIRED for six-membered rings. Simple linear alkyl chains at the C3 position were well tolerated, affording excellent conversions (>94%) to the expected products (**3a**–**3c**) with excellent diastereo- and enantioselectivity (>98:2 *dr* and 99% *ee*). A preparative scale reaction at 0.3 mmol gave **3a** with conversions of 93% and 41% isolated yield.

**Figure 1:**
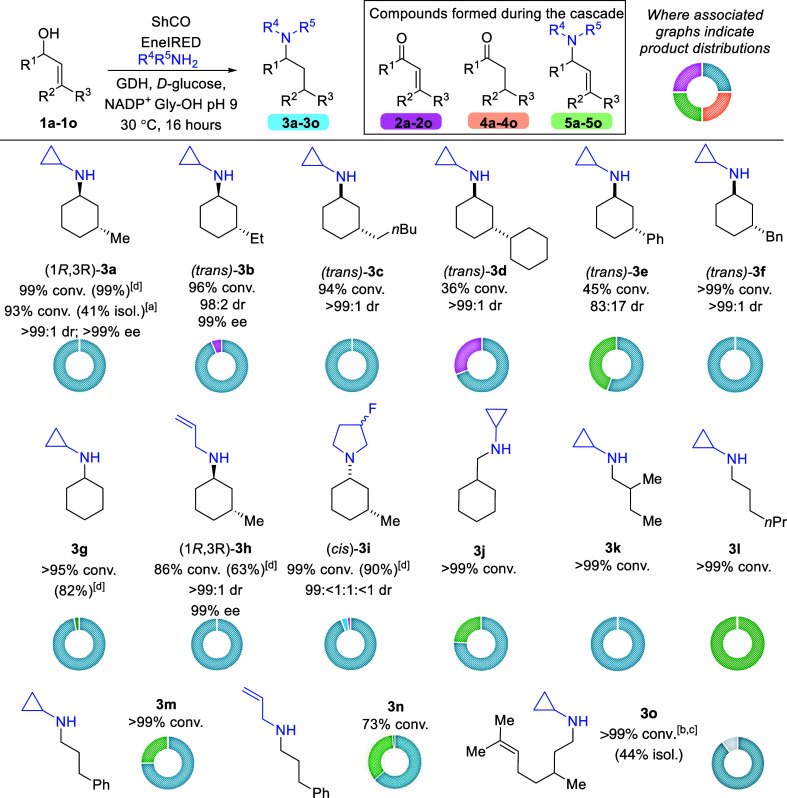
Scope of the ShCOa and eneIRED cascade. Desired amine products (**3a**–**3o**) are drawn, with product distributions illustrated by the donut chart. Conversions and product distributions are calculated based on percentage area from GCMS chromatograms against authentic analytical standards. [a] Conversions and yields for 0.3 mmol scale up. [b] *ee* not measured. [c] Aldimine intermediate observed (see the Supporting Information). [d] GCMS yield based on calibration curves (Supporting Information). Reaction conditions: purified ShCOa (1.5 mg/mL), EneIRED crude lysate (4 mg/mL), allylic alcohol (10 mM), amine (200 mM), GDH (1 mg/mL), D-glucose (10 mg/mL), NADP^+^ (1 mg/mL), 5% v/v DMSO, Gly-OH buffer corrected to pH 9. Total reaction volume = 500 μL. Reactions were shaken at 30 °C for 16 h. See the Experimental Section for further information.

To investigate steric effects at the 3-position, we introduced a cyclohexyl substituent. The oxidase-only transformation of substrate **1d** exhibited low conversion, a trend that was similarly reflected in the full cascade process. The second step of the cascade also proceeded slowly, with substantial intermediate enone **2d** observed, consistent with observations from the isolated EneIRED reaction.^7^ In contrast, conversions to desired **3e** reached 25% when a phenyl ring was introduced at this position (allylic alcohol **1e**) and intermediate enone **2e** was not detected. Interestingly the EneIRED-only reaction afforded only 13% conversion to **3e** from enone **2e**, suggesting that the cascade may enhance turnover by controlling the concentration of enone **2e** *in situ*. Furthermore, the cascade reaction yielded a higher proportion of the competing RA product **5e** compared with the EneIRED-only reaction, highlighting the influence of cascade dynamics on chemoselectivity. With a benzyl substituent at this position (allylic alcohol **1f**), conversions of >99% to the desired amine product **3f** were observed with excellent diastereoselectivity (>99:1). This represents a significant improvement over the EneIRED reaction alone from enone **2f**, where a large amount of ketone intermediate was seen (3:1:1, **4f**:**3f**:**2f**).^7^ Diastereoselectivity was also improved in the cascade compared with the EneIRED reaction alone. Unsubstituted cyclohexen-1-ol (**1g**) yielded a 95% conversion with 92% selectivity for the desired product (**3g**) and 8% RA product (**5g**) observed.

Finally, we screened both allylamine and 3-fluoropyrrolidine, which gave the desired products **3h** and **3i** in very good to excellent conversions (86% and 99%, respectively), with excellent diastereoselectivity and enantioselectivity (99% for **3h**, *ee* not determined for **3i**).

We then turned our attention to simple allylic alcohols that generate enal intermediates. We first examined simple disubstituted allylic alcohols, which delivered the desired amine products with >99% conversion to **3k**. From previous reports of the EneIRED only transformation from enal **2k**, the enantioselectivity with this substrate was poor (18% *ee*);^7^ therefore, this was not measured here. Submitting *E*-cinnamyl alcohol **1l** to the reaction conditions yielded both the CR-RA and RA products in a 3:1 ratio, respectively (**3l**:**5l**). A similar product distribution was observed when utilizing allylamine **3m**.

To investigate the influence of alkene geometry on the reaction outcome, the geometric isomers geraniol (*E*-isomer) and nerol (*Z*-isomer) were subjected to the reaction conditions. Nerol exhibited high reactivity, affording >99% conversion with the major product being the desired amine **3o** with approximately 10% of the aldimine intermediate observed (see the Supporting Information). Geraniol also underwent complete consumption (97%), but the predominant product was the aldimine intermediate, indicating that the reaction likely stalls at this stage, possibly due to the geometry and sterics impeding hydride delivery. Similar behavior has been observed in other biocatalytic cascade reactions involving this substrate.^18^ Allylic alcohols bearing a six-membered ring in which an enal is formed *in situ* (**1j**) proceeded in excellent conversion (>99%) and produced the desired amine and competitive RA product in a ratio of 3:1 (**3j**:**4j**).

Finally, we carried out a time-course analysis of the reaction to determine the changes in product distribution (Figure 2b). Key intermediates in this analysis were detected, including the enone **2a**, ketone **4a** resulting from the conjugate reduction, and the final amine product **3a** (Figure 2a).

**Figure 2:**
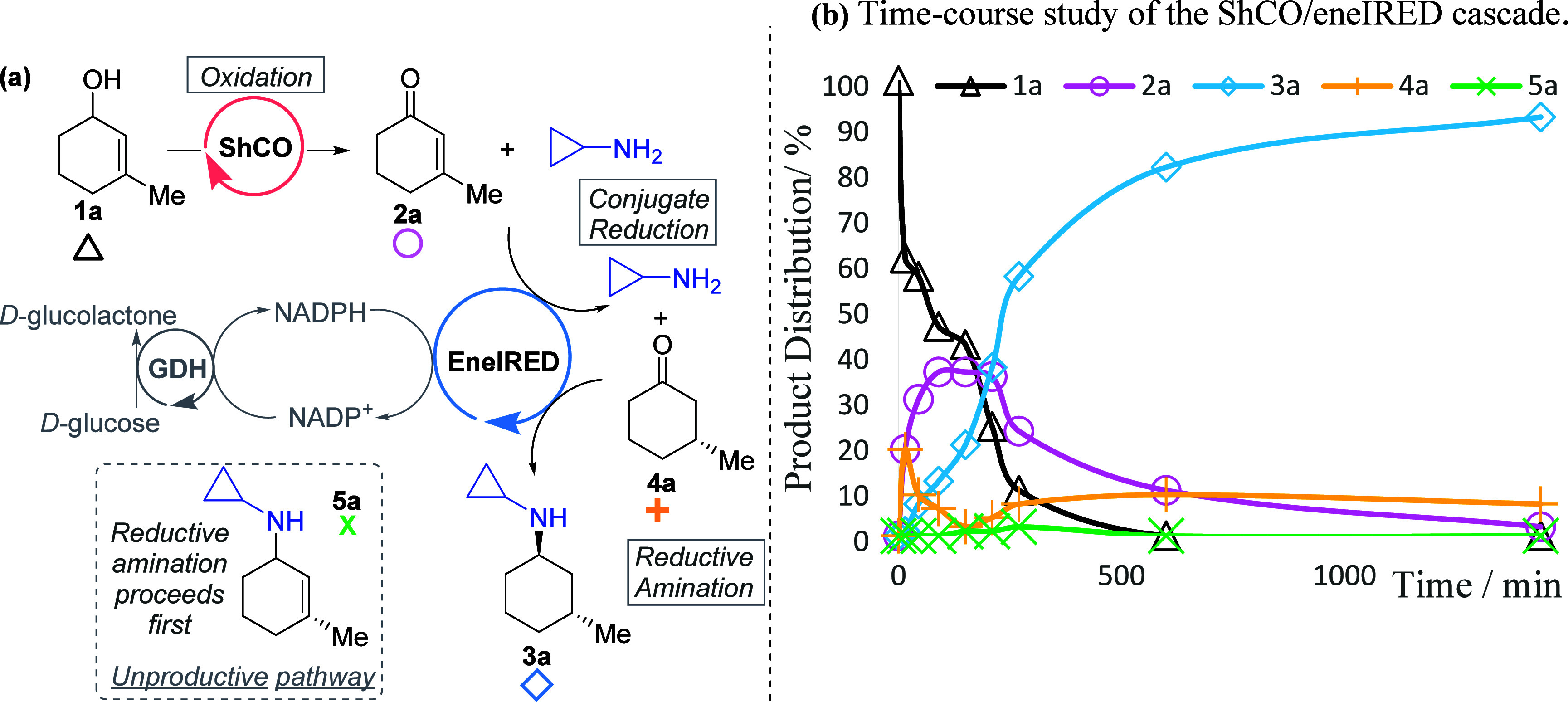
(a) Proposed reaction pathway; (b) time course study where conversions and product distributions are calculated based on percentage area from GCMS chromatograms against authentic analytical standards.

## 3 Conclusions

In conclusion, we have successfully developed a tandem enzymatic cascade in which allylic alcohols are oxidized *in situ*, followed by a conjugate reduction–reductive amination (CR-RA) sequence. In some examples, distinct differences in reaction profiles were observed depending on whether the full cascade process was employed or the EneIRED step was performed in isolation from the oxidase. These findings underscore the advantages of tandem enzymatic processes, which not only streamline multistep transformations into a single reaction vessel but often deliver improved outcomes compared to stepwise procedures.

## 4 Experimental Section

### 4.1 General Procedure for Analytical Scale Biotransformations for the ShCO_a_ Oxidations from Scheme 2 (500 μL Total Volume)

The following stock solutions were used in the setup of these reactions: ShCO_a_ stock concentrations range between 7 and 12 mg/mL stored in 100 mM phosphate buffer, pH 7.4. Allylic alcohol: stock concentration of 1 M in DMSO. Glycine buffer = 100 mM corrected to pH 9.

Unless stated otherwise, the following components were added to a 1.5 mL microcentrifuge tube in the following order: glycine buffer (final volume = 500 μL), DMSO (15 μL), allylic alcohol stock solution (10 μL, final concentration = 10 mM), and ShCO_a_ (final concentration = 1.5 mg/mL). The reaction mixture was shaken at 200 rpm and 30 °C for 16 h. The reaction mixture was extracted with EtOAc (2 × 500 μL) followed by centrifugations, and the organic extractions were used directly for GC-FID and/or GCMS analysis.

### 4.2 General Procedure for Analytical Scale Biotransformations for the ShCO_a_–EneIRED Cascade Shown in Figure 1 (500 μL Total Volume)

The following stock solutions were used in the setup of these reactions: ShCO_a_ stock concentrations range between 7 and 12 mg/mL stored in 100 mM phosphate buffer, pH 7.4. EneIRED stock concentration = 40 mg/mL in 100 mM phosphate buffer, pH 7.4. Allylic alcohol stock concentration = 1 M in DMSO. NADP^+^ stock concentration = 10 mg/mL. D-Glucose stock concentration was 100 mg/mL in dH_2_O. GDH stock concentration = 10 mg/mL in 100 mM phosphate buffer pH 7.4. amine (stock concentration of 500 mM in glycine buffer corrected to pH 9). Glycine buffer = 100 mM corrected to pH 9.

Unless stated otherwise, the following components were added to a 1.5 mL microcentrifuge tube in the following order: glycine buffer (final volume = 500 μL), NADP^+^ stock solution (50 μL, final concentration = 1 mg/mL), D-glucose stock solution (50 μL, final concentration = 10 mg/mL), GDH stock solution (50 μL, final concentration = 1 mg/mL), DMSO (15 μL), amine stock solution (200 μL, 20 equiv., final concentration = 200 mM), EneIRED stock solution (50 μL, final concentration = 4 mg/mL), ShCO_a_ stock solution (final concentration = 1.5 mg/mL), and allylic alcohol stock solution (10 μL, final concentration = 10 mM). The reaction mixture was shaken at 200 rpm and 30 °C for 16 h. The reactions were terminated by addition of 5.0 M NaOH (aq. 100 μL), clarified by centrifugation, and extracted into EtOAc (2 × 500 μL) followed by further centrifugation. The organic extractions were used directly for GC-FID or GCMS analysis.

### 4.3 General Procedure for Scale Up Procedures for the ShCO_a_–EneIRED Cascade (0.3 mmol Scale)

The following stock solutions were used in the setup of these reactions: ShCO_a_ stock concentration = 10 mg/mL stored in 100 mM phosphate buffer, pH 7.4. EneIRED stock concentration = 40 mg/mL in 100 mM glycine buffer, pH 9.0. Allylic alcohol stock concentration = 1 M in DMSO. NADP^+^ stock concentration = 10 mg/mL. D-Glucose stock concentration = 100 mg/mL in dH_2_O. GDH stock concentration = 2 mg/mL in glycine buffer, pH 9.0. Amine stock concentration = 500 mM in glycine buffer corrected to pH 9. Glycine buffer = 100 mM corrected to pH 9.

To a 50 mL Falcon tube, the following components were added in the following order: NADP^+^ stock solution (3.0 mL, final concentration = 1 mg/mL), D-glucose stock solution (3.0 mL, final concentration = 10 mg/mL), amine stock solution (12 mL, 20 equiv., final concentration = 200 mM), and glycine buffer (1 mL, 100 mM, pH 9.0). The resulting solution was readjusted to pH 9.0. DMSO was then added (1.2 mL, final concentration = 5% v/v), followed by the addition of stock solutions of GDH (1.5 mL, final concentration = 0.1 mg/mL), EneIRED (3.0 mL, final concentration 4 mg/mL), and ShCO_a_ (4.5 mL, final concentration = 1.5 mg/mL). Finally, allylic alcohol stock solution (300 μL, final concentration = 10 mM) was added to the reaction mixture, and the reaction volume was made up to 30 mL with glycine buffer, before shaking in an incubator (48 h, 200 rpm, 30 °C). Extraction with Et_2_O (3 × 10 mL) and centrifugation were used to separate layers effectively. The organic fractions were then washed with deionized water (4 × 10 mL) and dried (MgSO_4_), and the solvent was removed *in vacuo*, yielding the crude product.

## Supplementary Material



## Notes

Materials, enzyme expression, detailed experimental procedures, analytical methods, GC spectra, ^1^H and ^13^C NMR spectra (PDF)

## References

[1] Nugent, T. C. Chiral Amine Synthesis. Methods, Developments and Applications; Wiley-VCH: Weinheim, 2010.

[2] Afanasyev O. I., Kuchuk E., Usanov D. L., Chusov D. (2019). Reductive
Amination
in the Synthesis of Pharmaceuticals. Chem. Rev..

[3] Bell E. L., Finnigan W., France S. P., Green A. P., Hayes M. A., Hepworth L. J., Lovelock S. L., Niikura H., Osuna S., Romero E., Ryan K. S., Turner N. J., Flitsch S. L. (2021). Biocatalysis. Nature Reviews Methods Primers.

[4] Yuan B., Yang D., Qu G., Turner N. J., Sun Z. (2024). Biocatalytic
Reductive Aminations with NAD­(P)­H-Dependent Enzymes: Enzyme Discovery,
Engineering and Synthetic Applications. Chem.
Soc. Rev..

[5] Lewis R. D., France S. P., Martinez C. A. (2023). Emerging Technologies for Biocatalysis
in the Pharmaceutical Industry. ACS Catal..

[6] Marshall J. R., Yao P., Montgomery S. L., Finnigan J. D., Thorpe T. W., Palmer R. B., Mangas-Sanchez J., Duncan R. A. M., Heath R. S., Graham K. M., Cook D. J., Charnock S. J., Turner N. J. (2021). Screening
and Characterization of a Diverse Panel of Metagenomic Imine Reductases
for Biocatalytic Reductive Amination. Nat. Chem..

[7] Thorpe T. W., Marshall J. R., Harawa V., Ruscoe R. E., Cuetos A., Finnigan J. D., Angelastro A., Heath R. S., Parmeggiani F., Charnock S. J., Howard R. M., Kumar R., Daniels D. S. B., Grogan G., Turner N. J. (2022). Multifunctional Biocatalyst for Conjugate
Reduction and Reductive Amination. Nature.

[8] Rosenthal K., Bornscheuer U. T., Lütz S. (2022). Cascades of
Evolved Enzymes for the
Synthesis of Complex Molecules. Angew. Chem.,
Int. Ed..

[9] Muschiol J., Peters C., Oberleitner N., Mihovilovic M. D., Bornscheuer U. T., Rudroff F. (2015). Cascade Catalysis – Strategies
and Challenges En Route to Preparative Synthetic Biology. Chem. Commun..

[10] Huffman M. A., Fryszkowska A., Alvizo O., Borra-Garske M., Campos K. R., Canada K. A., Devine P. N., Duan D., Forstater J. H., Grosser S. T., Halsey H. M., Hughes G. J., Jo J., Joyce L. A., Kolev J. N., Liang J., Maloney K. M., Mann B. F., Marshall N. M., McLaughlin M., Moore J. C., Murphy G. S., Nawrat C. C., Nazor J., Novick S., Patel N. R., Rodriguez-Granillo A., Robaire S. A., Sherer E. C., Truppo M. D., Whittaker A. M., Verma D., Xiao L., Xu Y., Yang H. (2019). Design of
an in Vitro Biocatalytic Cascade for the Manufacture of Islatravir. Science (1979).

[11] Ricca E., Brucher B., Schrittwieser J. H. (2011). Multi-Enzymatic
Cascade Reactions:
Overview and Perspectives. Adv. Synth Catal.

[12] Sangster J. J., Ruscoe R. E., Cosgrove S. C., Mangas-Sánchez J., Turner N. J. (2023). One-Pot Chemoenzymatic Cascade for the Enantioselective
C(1)-Allylation of Tetrahydroisoquinolines. J. Am. Chem. Soc..

[13] Harawa V., Thorpe T. W., Marshall J. R., Sangster J. J., Gilio A. K., Pirvu L., Heath R. S., Angelastro A., Finnigan J. D., Charnock S. J., Nafie J. W., Grogan G., Whitehead R. C., Turner N. J. (2022). Synthesis of Stereoenriched
Piperidines
via Chemo-Enzymatic Dearomatization of Activated Pyridines. J. Am. Chem. Soc..

[14] Ford G. J., Swanson C. R., Bradshaw
Allen R. T., Marshall J. R., Mattey A. P., Turner N. J., Clapés P., Flitsch S. L. (2022). Three-Component
Stereoselective Enzymatic Synthesis of Amino-Diols and Amino-Polyols. JACS Au.

[15] Lenz M., Borlinghaus N., Weinmann L., Nestl B. M. (2017). Recent Advances
in Imine Reductase-Catalyzed Reactions. World
J. Microbiol. Biotechnol..

[16] Thorpe T. W., France S. P., Hussain S., Marshall J. R., Zawodny W., Mangas-Sanchez J., Montgomery S. L., Howard R. M., Daniels D. S. B., Kumar R., Parmeggiani F., Turner N. J. (2019). One-Pot Biocatalytic
Cascade Reduction of Cyclic Enimines for the Preparation of Diastereomerically
Enriched *N* -Heterocycles. J.
Am. Chem. Soc..

[17] Al-Shameri A., Borlinghaus N., Weinmann L., Scheller P. N., Nestl B. M., Lauterbach L. (2019). Synthesis
of *N* -Heterocycles from
Diamines *via* H _2_ -Driven NADPH Recycling
in the Presence of O _2_. Green Chem..

[18] Ramsden J.
I., Heath R. S., Derrington S. R., Montgomery S. L., Mangas-Sanchez J., Mulholland K. R., Turner N. J. (2019). Biocatalytic N-Alkylation
of Amines Using Either Primary Alcohols or Carboxylic Acids via Reductive
Aminase Cascades. J. Am. Chem. Soc..

[19] Chen H., Zhou Z., Kong W. (2021). Allylic Alcohol Synthesis by Ni-Catalyzed
Direct and Selective Coupling of Alkynes and Methanol. Chem. Sci..

[20] Lumbroso A., Cooke M. L., Breit B. (2013). Catalytic
Asymmetric Synthesis of
Allylic Alcohols and Derivatives and Their Applications in Organic
Synthesis. Angewandte Chemie - International
Edition.

[21] Heath R. S., Sangster J. J., Turner N. J. (2022). An Engineered Cholesterol Oxidase
Catalyses Enantioselective Oxidation of Non-Steroidal Secondary Alcohols. ChemBioChem..

