# Supplementary material for: Alcohol Oxidase–Imine Reductase Cascade for One-Pot Chiral Amine Synthesis
Source: ACS Catal. 2026 Jan 14;16(3):2119–23. doi: 10.1021/acscatal.5c06313 (PMC12888005; doi:10.1021/acscatal.5c06313)
Supplement: Supplementary file 1 [file cs5c06313_si_001.pdf]

Alcohol Oxidase–Imine Reductase Cascade for One-Pot Chiral Amine Synthesis

Supplementary Information

Anya Miletic,[†] *Christopher J. Truby*,[‡] Nicholas J. Turner[†] and Rebecca E. Ruscoe[*,‡]

Corresponding author email address: r.ruscoe@keele.ac.uk

[†]Department of Chemistry, University of Manchester, Manchester Institute of Biotechnology, 131 Princess Street, Manchester M1 7DN, U.K.
[‡]School of Chemical and Physical Sciences & School of Allied Health Professions and Pharmacy, Keele University, Keele, Staffordshire ST5 5BG, U.K.

Contents

1.0 Experimental Procedures 3
1.1 General 3
1.2 Gas Chromatography Analysis 3
1.2.1 GC-FID 3
1.2.2 GCMS 3
1.3 Biocatalysts 3
1.3.1 Protein sequences 3
1.3.2 Protein Purification 4
2.0 Synthesis of Substrates and Analytical Standards 5
2.1 General Procedures 5
General Procedure A – Synthesis of allylic alcohols 5
General Procedure B – Synthesis of secondary amines 5
General Procedure C – Synthesis of ketones 6
General Procedure D – Synthesis of allylic amines 6
2.2 Allylic Alcohol Starting Materials 6
(1-Cyclohexenyl)methanol (1j) 6
3-Ethyl-2-cyclohexen-1-ol (1b) 7
3-Pentylcyclohex-2-en-1-ol (1c) 7
3-Cyclohexyl-cyclohex-2-en-1-ol (1d) 7
3-Phenylcyclohex-2-en-1-ol (1e) 8
3-Benzylcyclohex-2-en-1-ol (1f) 8
2.2 Enals and Enones 9
2.3 Aldehydes and Ketones 9
3-Cyclohexylcyclohexanone (4d) 9
3-Phenylcyclohexanone (4e) 9
2.4 Intermediate allylic amines 10
N-(3,7-Dimethylocta-2,6-dien-1-yl)cyclopropanamine (5o) 10
2.5 Synthesis of racemic saturated amines 10
N-Cyclopropylcyclohexylamine (3g) 11
rac-N-Cyclopropylcitronellamine (3o) 11
rac-N-Cyclopropyl[3-bi(cyclohexyl)yl]amine (3d) 12
Enantiomerically Enriched Products 12
3.0 Biotransformations 14
3.1 General procedure for analytical scale biotransformations for the ShCOa oxidations (500 µL total volume) 14
3.2 Overview of allylic alcohols screened with ShCO 14
3.3 General procedure for analytical scale biotransformations for the ShCO – EneIRED cascade (500 µL total volume) 15
3.4 General procedure for scale up procedures for ShCO – EneIRED cascade (0.3 mmol scale) 15
3.4.1 Biocatalytic scale up to form *N*-cyclopropyl-3-methylcyclohexa-1-amine (3a) 15
3.4.2 Biocatalytic scale up to form N-cyclopropyl-3-methylcyclohexa-1-amine (3o) 17
4.0 Gas Chromatography (GC) analysis of biotransformations 18

4.1 GC-FID Traces from optimisation of ShCOa catalysed oxidations shown in Table 1 with further examples shown. 18
4.2 GCMS/GC Traces from biotransformations shown in Scheme 2 (Oxidation only) 21
4.2 GCMS Traces from biotransformations (full cascade) shown in Scheme 3....23
4.3 Chiral GC analysis 27
4.4 GCMS Time course data 31
4.3 Product Distributions from GCMS Data in Table Format 33
4.4 GCMS Calibration 34
5.0 References 37
6.0 NMR Spectra of New Compounds 38

1.0 Experimental Procedures

1.1 General

Unless stated otherwise all chemicals were purchased from and used directly from commercial suppliers without in-house purification (Sigma-Aldrich, Merck, Alfa Aesar and Acros Organics and Fisher). GC gases were obtained from BOC gases (Guildford, UK). Small molecule NMR spectroscopy was recorded using a Bruker Avance or Bruker Ascend (^{1}H at 400 MHz, ^{13}C at 100 MHz, ^{19}F 275 MHz), *J* values are given in Hz and reported chemical shifts (δ, ppm) are relative to the deuterated solvent residual protic signal. High-resolution mass spectrometry (HRMS) was recorded using a Waters LCT time-of-flight mass spectrometer, connected to a Waters Alliance LC (Waters, Milford, MA, USA). Agilent Masshunter software was used for the data processing.

1.2 Gas Chromatography Analysis

1.2.1 GC-FID

GC-FID analysis was performed using an Agilent 8860 series gas chromatograph, at constant He flow with flame ionisation detector (FID).

GC-FID-rac

Racemic analysis was carried out using an Agilent HP-5 column (30 m, 0.32 mm, 0.25 μm) with the following method: 70 °C to 200 °C at 20 °C/min 1.2 mL/min, inlet 250 °C, detector 300 °C. Split mode with split ratio 100:1 and split flow 650 mL/min.

GC-FID-chiral

Chiral GC analysis was preformed using *a* BetaDex325 column (30 m × 0.32 mm × 0.25 μm). 1.2 mL/min, 50 °C to 200 °C at 2 °C/min, inlet 200 °C, detector 250 °C.

1.2.2 GCMS

GCMS analysis was performed using one of the following methods:

GCMS-A

Using an Agilent 7890B Series GC with 5977B MS-EI detector in positive mode at a constant He flow. *Column*: Agilent HP-1MS column (30 m × 0.32 mm × 0.25 μm). 40 °C to 300 °C at 30 °C/min and 1mL/min, injector 270 °C, transfer line 320°C, quadrupole 150 °C, ion source 230 °C.

GCMS-B

Using an Agilent 8890 Series GC with 5977B GC/MSD detector in positive mode at a constant He flow. *Column*: Agilent HP-5MS UI column (30 m × 250 μm × 0.25 μm). 40 °C to 300 °C at 20 °C/min and 1 mL/min, injector 270 °C, transfer line 320 °C, quadrupole 150 °C, ion source 230 °C. Split mode with split ratio 10:1 and split flow 10 mL/min.

1.3 Biocatalysts

Glucose dehydrogenase CDX-901 was purchased from Codexis, US. Plasmids for ShCO and EneIRED were supplied by the Turner group. ShCO mutants were produced in house according to the reported literature procedure,[1] and purified before use. The eneIRED utilised in this work was produced in house according to the literature procedure and used as cell free extracts (CFE).[2]

1.3.1 Protein sequences

Streptomyces hygrospinosus Cholesterol Oxidase (ShCO)
MFENQQNQHLSRRRLLGLAALSGAAVTGLTTISAAPRAAAADKRSPRADSGSFVPAVVIGTGYGAAVSALR
LGEAGVETLMLEMGQLWNKPAEDGNVFCGMLTPDRRSSWFKSRTEAPLGSFLWLDVINRDIEPYAGVLDR
VHFDQMSVYVGRGVGGGSLVNGGMAVVPKRAYFEEVLPQVDAAQMYERYFPRANAALKVNHIDPAWFE
KTEWYNFARVSREQAGKAGLSTTFVPNVYDFDHMQREAAGTAPKSALAGEVIYGNNHGKQSLDKTYLAAA
LGTGKVTIETLHRVTAIRQQADGSYVLSVDQSDANGTVIAHKEIACRHLFLGAGSLGSTELLVRARDTGALP
HLNAEVGEGWGPNGNIMTGRANHVWNPTGAHQSSIPALGIDDWDNPDAPVFAEIAPMPAGLETWVSLYLA
ITKNPQRGSFVYDKATDRAMLRWTREQNAPAVAAAKSLFDRINKANTTMYRYDLFGPQLKNFADDFCYHP
LGGCVLGKATDDYGRVAGYHNLYVTDGALIPGSIGVNPFVTITALAERNIERVIAEDVRTAA

Mutant ShCOa was used in all experiments, unless stated otherwise.
ShCOa = E404C/P409S
ShCOb = E404A/P409I

EneIRED

MSMSGSNKPSVSVLGLGAMGSVLARTLLQAGYGVTVWNRSPERATALVQEGASLAREASEAINASNLIIIC
MIDKAVFQDVLSSLDPLLNMSGKTIVNMSTGTVDDVERIAKRVDQHNGLYVDAGIMCYPKDIGGQHTTILYS
GNSDAYHAHESTLKVLAGNPKFLGADPTACTPTYLALYAFYFGAFAAWLEGAVLASCAGVSVQDFKALSPI
MSDMLVDGIKTAADRIAASDYSGEQASVDVHVAGQEVVLDALQRANAPHASTDAYLSYCRMAQTAGMGE
LDIASLFKAMHP

1.3.2 Protein Purification

ShCO

ShCO was purified using immobilized metal affinity chromatography (IMAC). The column was equilibrated with 100 mM potassium phosphate buffer pH 7 (5 x CV) before being loaded with filtered (using 0.4 uM sterile filters) CFE suspended in 100 mM KPi buffer pH 7. The bound protein was washed with 100 mM potassium phosphate (3 x CV), followed by elution using Buffer A (100 mM potassium phosphate, 20 mM imidazole, 300 mM NaCl, pH 7) (5 x CV) and Buffer B (100 mM potassium phosphate, 500 mM imidazole, 100 mM NaCl, pH 7) (5 x CV). Eluted fractions were analysed by SDS-PAGE (Figure S1), where fractions containing sufficiently pure protein were combined and concentrated using centrifugation (4000 RPM, 15 °C, 10 kDa MW cut-off). The protein concentrate was exchanged into 100 mM potassium phosphate buffer, pH 7, using a Vivaspin (Cytiva) 10 kDa MWCO centrifugal concentrator. Protein concentration was measured using NanoDrop™ 2000 Spectrophotometer (Thermofisher) using protein A280 function. The catalyst preparation could be kept at 4 °C for short term (days) storage or after flash freezing at -78 °C for long term storage (months).

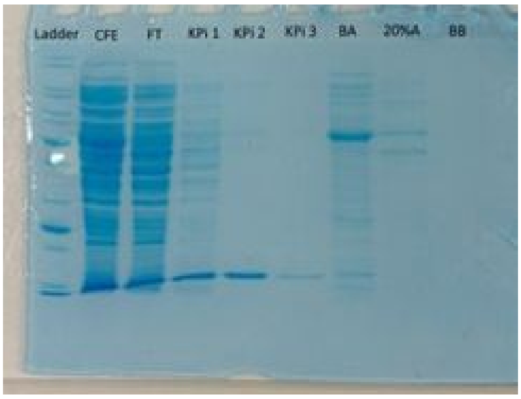

***Figure S1**: SDS-PAGE gel from ShCOa purification. Lane 1 = ladder, lane 2 = cell free extract (CFE), lane 3 = flow through, lane 4 = 1st column volume KPi wash, lane 5 = 2nd column volume KPi wash, lane 6 = 3rd column volume KPi wash, lane 7 = buffer A wash, lane 8 = 20% buffer B in buffer A and lane 9 = buffer B wash.*

EneIRED

Cells expressing EneIRED were lysed using sonication and centrifugation to remove cell debris. The cell free extract was lyophilized and re-suspended in 100 mM KPi to form a stock solution with 40 mg/mL of CFE for biocatalytic reactions.

2.0 Synthesis of Substrates and Analytical Standards

2.1 General Procedures

General Procedure A – Synthesis of allylic alcohols

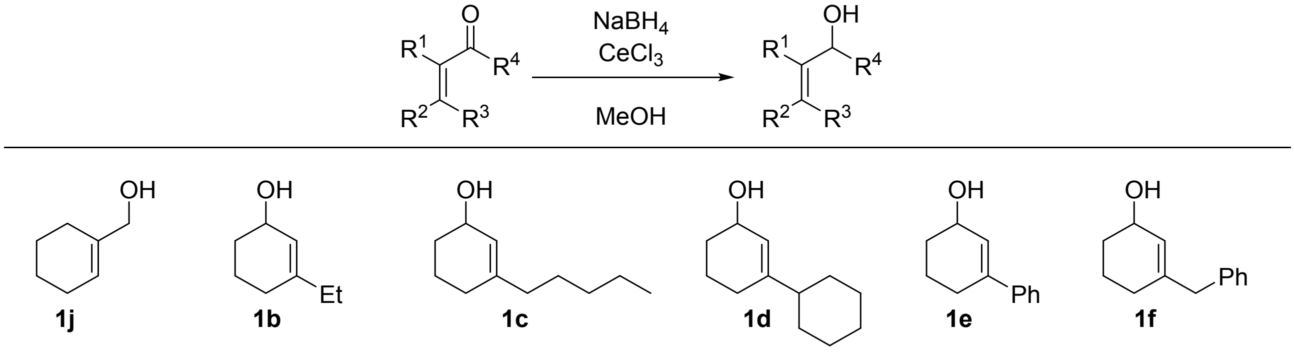

$CeCl_3$ (247 mg, 1.0 mmol, 1.0 equiv.) was added to MeOH (2 mL) and stirred at room temperature for 5 minutes. Enone/Enal (1.0 mmol, 1.0 equiv.) was added, followed by the addition of $NaBH_4$ (76 mg, 2.0 mmol, 2.0 equiv.) and stirred for 2 hours. The reaction was quenched by the slow addition of H_2O (5 mL), followed by extraction with Et_2O (3 × 5 mL). The combined organic layers were dried ($MgSO_4$) and solvent was removed *in vacuo*.

General Procedure B – Synthesis of secondary amines

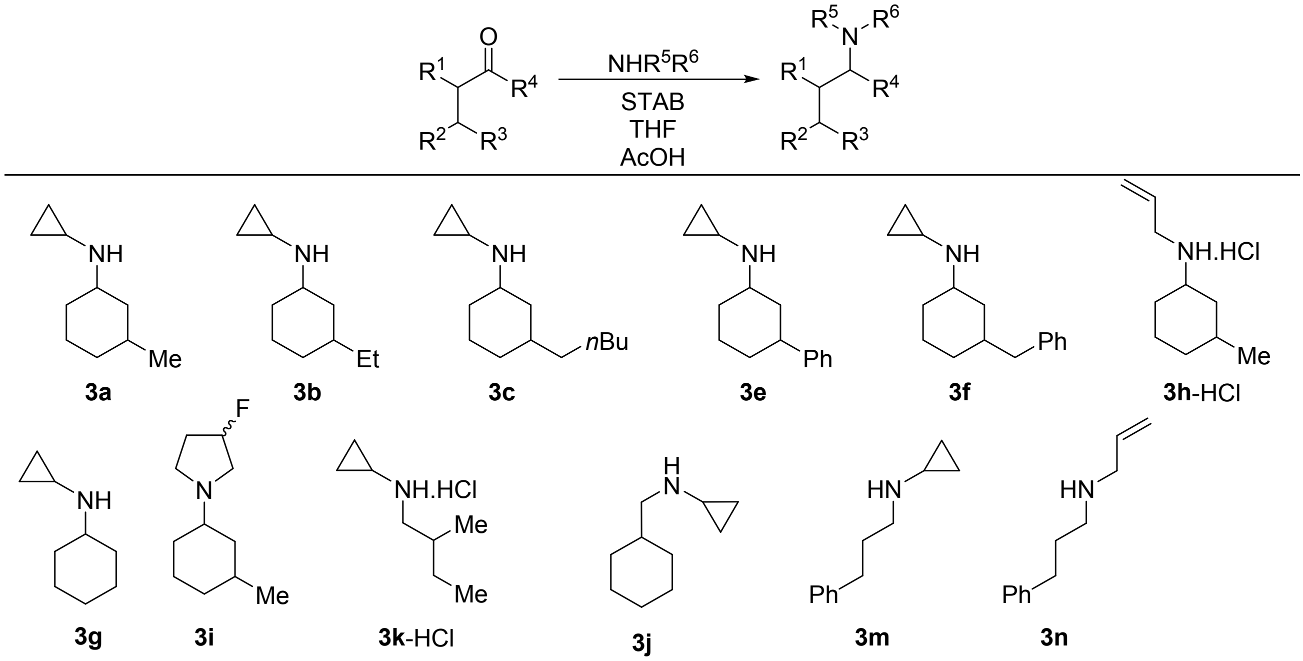

To a solution of the ketone or aldehyde (1.0 equiv.) in dry THF (0.2 M = reaction conc.) were added the corresponding amine (1.1 equiv.), sodium triacetoxyborohydride (1.5 equiv.) and glacial acetic acid (1 equiv.). The reaction was stirred overnight at room temperature before being quenched with aqueous $NaHCO_3$ (sat., 1 × reaction volume) and extracted into Et_2O (2 × reaction volume). The organic fractions were combined and then extracted with aqueous HCl (1 M, 3 × reaction volume). The acidic aqueous fractions were then combined before being adjusted to pH 12 with aqueous NaOH (5 M) and extracted with Et_2O (3 × reaction volume). The organic fractions were combined, dried with magnesium sulphate, filtered and then concentrated *in vacuo* to yield the corresponding secondary amines.

General Procedure C – Synthesis of ketones

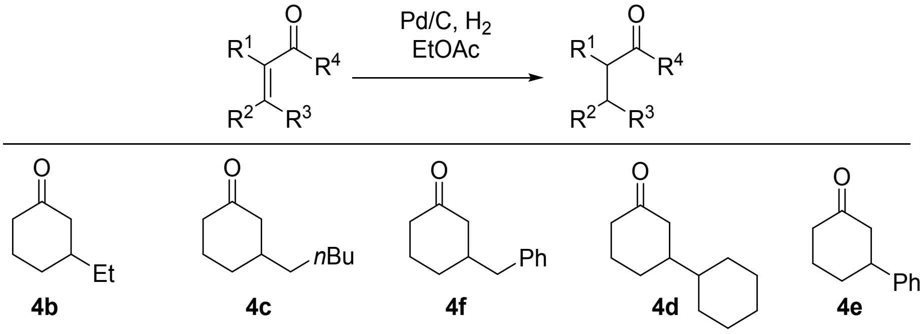

To a solution of enal/enone (1.0 mmol, 1.0 equiv.) in EtOAc (1.5 mL) was added 10% Pd/C (0.05 mmol, 5 mol%). The flask was evacuated and refilled with N_2 three times, before a balloon of H_2 was bubbled through the suspension for 30 minutes. The suspension was stirred at room temperature under a H_2 atmosphere for 18 hours. Filtration over Celite®, followed by concentration under reduced pressure gave the crude product, which was purified by column chromatography.

General Procedure D – Synthesis of allylic amines

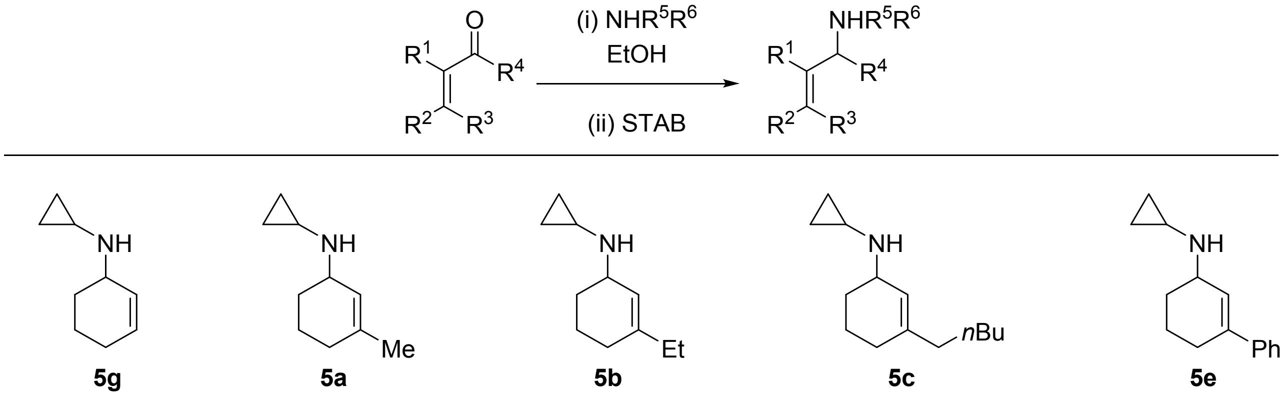

The corresponding amine was added to a solution of enone (1.0 equiv.) in ethanol (0.25 M = reaction conc.) and was stirred at room temperature for 16 h. The reaction mixture was cooled to 0 °C before the addition of sodium triacetoxyborohydride (4.4 eq). The reaction was allowed to warm to room temperature after 10 min. After stirring for 16 h, the reaction was quenched by the addition of water (1 × reaction volume), extracted with ethyl acetate (3 × reaction volume), dried with magnesium sulphate, filtered and then concentrated *in vacuo* to give the corresponding enamines.

2.2 Allylic Alcohol Starting Materials

The following compounds are commercially available and were purchased from either Fluorochem, Fisher Scientific or Merck: 2-Cyclohexen-1-ol, (2*E*)-Hexen-1-ol, 3-Methyl-2-cyclohexen-1-ol, (*E*)-2-Methyl-2-buten-1-ol.

(1-Cyclohexenyl)methanol (1j)

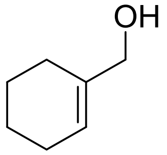

1j

1-Cyclohexene-1-carboxaldehyde (0.22 mL, 2.0 mmol, 1.0 equiv.) was added dropwise to a solution of $LiAlH_4$ (84 mg, 2.2 mmol, 1.1 equiv.) in anhydrous THF (15 mL) at room temperature under an atmosphere of nitrogen. The reaction mixture was stirred for 1 hour at room temperature before being poured onto ice-cold water 20 mL), extracted with EtOAc (3 × 20 mL), dried ($MgSO_4$), filtered and concentrated under reduced pressure to give the titled compound (220 mg, 1.96 mmol, 98%) as a colourless oil, which required no further purification.

NMR Data
^{1}H NMR (400 MHz, MeOD) δ 5.68 – 5.61 (1H, m, =CH), 3.88 (2H, s, CH_2OH), 2.07 – 1.95 (4H, m, CH_2), 1.70 – 1.54 (4H, m, CH_2); ^{13}C NMR (101 MHz, MeOD) δ 138.7 (C=), 123.5 (=CH), 67.7 (CH_2OH), 26.6 (CH_2), 26.0 (CH_2), 23.8 (CH_2), 23.7 (CH_2). Data consistent with literature reports.[3]

3-Ethyl-2-cyclohexen-1-ol (1b)

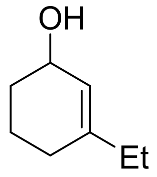

1b

Prepared according to *General Procedure A*. 3-Ethylcyclohex-2-en-1-one (252 mg, 2.0 mmol, 1.0 equiv.), $CeCl_3$ (494 mg, 1.0 mmol, 1.0 equiv.) and $NaBH_4$ (152 mg, 4.0 mmol, 2.0 equiv.) in MeOH (10 mL) gave the titled compound (79 mg, 0.62 mmol, 31%), which required no further purification.

NMR Data
^{1}H NMR (400 MHz, $CDCl_3$) δ 5.49 (1H, dt, J = 3.5, 1.7 Hz, CH), 4.24 – 4.16 (1H, m, CH), 2.04 – 1.88 (4H, m, CH_2), 1.83 – 1.69 (2H, m, CH_2), 1.64 – 1.42 (3H, m, OH, CH_2), 1.01 (3H, t, J = 7.4 Hz, CH_3); ^{13}C NMR (101 MHz, $CDCl_3$) δ 144.2 (C), 122.5 (CH), 66.1 (CH), 32.2 (CH_2), 30.3 (CH_2), 28.7 (CH_2), 19.3 (CH_2), 12.2 (CH_3). Data consistent with literature.[4]

3-Pentylcyclohex-2-en-1-ol (1c)

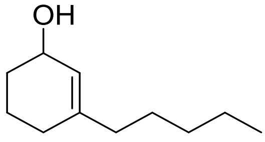

1c

Prepared according to *General Procedure A*. 3-Pentyl-2-cyclohexen-1-one (336 mg, 2.0 mmol, 1.0 equiv.), $CeCl_3$ (494 mg, 1.0 mmol, 1.0 equiv.) and $NaBH_4$ (152 mg, 4.0 mmol, 2.0 equiv.) in MeOH (10 mL). Purification over silica gel (20% EtOAc in petroleum ether) gave the titled compound (133 mg, 0.79 mmol, 40%) as a colourless oil.

NMR
^{1}H NMR (400 MHz, $CDCl_3$) δ 5.49 (1H, app. dp, J = 3.0, 1.5 Hz, CH), 4.19 (1H, app. br. s, CH), 2.00 – 1.86 (4H, m, CH_2), 1.82 – 1.67 (2H, m, CH_2), 1.63 – 1.52 (2H, m, CH_2), 1.45 – 1.20 (6H, m, CH_2), 0.88 (3H, t, J = 7.1 Hz, CH_3); ^{13}C NMR (101 MHz, $CDCl_3$) δ 142.9 (C), 123.7 (CH), 66.1 (CH), 37.7 (CH_2), 32.1 (CH_2), 31.7 (CH_2), 28.7 (CH_2), 27.3 (CH_2), 22.7 (CH_2), 19.3 (CH_2), 14.2 (CH_3).

IR
ν_{max}/cm^{-1} 3314 (OH), 2927 (CH), 2858 (CH), 1650 (C=C), 1456, 1377, 1342, 1285, 1160, 1060, 1032, 958, 909;

HRMS (ESI positive mode)
HRMS calcd. For $C_{11}H_{20}O$:168.1514. Found: 168.1522.

3-Cyclohexyl-cyclohex-2-en-1-ol (1d)

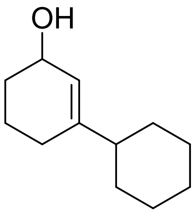

1d

Prepared according to *General Procedure A*. 3-Cyclohexylcyclohex-2-enone (29 mg, 0.16 mmol, 1.0 equiv.), $CeCl_3.7H_2O$ (61 mg, 0.16 mmol, 1.0 equiv.), $NaBH_4$ (6 mg, 0.32 mmol, 2.0 equiv.) in MeOH (2 mL). Purification over silica gel (20% EtOAc in petroleum ether) gave the titled compound (18 mg, 0.1 mmol, 62%) as a colourless oil.

<u>*NMR*</u>
^{1}H NMR (400 MHz, $CDCl_3$) δ 5.49 – 5.45 (1H, app. dq, *J* = 2.9, 1.5 Hz, CH), 4.22 – 4.16 (1H, m, CH), 2.01 – 1.89 (2H, m, CH_2), 1.84 – 1.63 (m, 5H, CH and CH_2), 1.62 – 1.52 (2H, m, CH_2), 1.33 – 1.08 (8H, m, CH_2); ^{13}C NMR (101 MHz, $CDCl_3$) δ 147.7 (C), 122.0 (CH), 66.2 (CH), 45.6 (CH), 32.3 (CH_2), 31.9 (CH_2), 31.9 (CH_2), 27.1 (CH_2), 26.8 (CH_2), 26.8 (CH_2), 26.5 (CH_2), 19.5 (CH_2).

<u>*IR*</u>
v_{max}/cm^{-1} 3366 (OH), 2922 (CH), 2850 (CH), 1725, 1660 (C=C), 1448, 1345, 1288, 1258, 1159, 1068, 969, 912, 892.

<u>*HRMS (ESI positive mode)*</u>
HRMS calcd. For $C_{12}H_{20}O(M+NH_4)^+$:198.1852. Found: 198.1824. Calcd. $(M+Na)^+$:203.1406. Found 203.1366.

3-Phenylcyclohex-2-en-1-ol (1e)

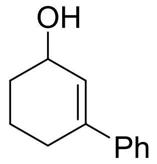

1e

Prepared according to general procedure A. 3-Phenylcyclohex-2-enone (172 mg, 1.0 mmol, 1.0 equiv.), $CeCl_3$ (247 mg, 1.0 mmol, 1.0 equiv.) and $NaBH_4$ (76 mg, 2.0 mmol, 2.0 equiv.) in MeOH (5 mL) gave the titled compound (76 mg, 0.44 mmol, 44%), which required no further purification.

<u>*NMR*</u>
^{1}H NMR (400 MHz, $CDCl_3$) δ 7.44 – 7.38 (2H, m, ArCH), 7.36 – 7.30 (2H, m, ArCH), 7.29 – 7.23 (1H, m, ArCH), 6.13 (1H, dt, *J* = 3.6, 1.8 Hz, CH), 4.39 (1H, s, CH), 2.55 – 2.30 (2H, m, CH_2), 2.06 – 1.84 (2H, m, CH_2), 1.79 – 1.62 (2H, m, CH_2), 1.54 (1H, s, OH); ^{13}C NMR (101 MHz, $CDCl_3$) δ 141.5 (C), 140.4 (CH, ArC), 128.5 (ArCH), 127.6 (ArCH), 126.7 (ArCH), 125.5 (ArCH), 66.5 (CH), 31.9 (CH_2), 27.7 (CH_2), 19.6 (CH_2). Data consistent with literature.[4]

3-Benzylcyclohex-2-en-1-ol (1f)

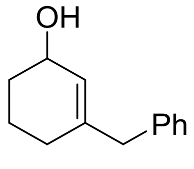

1f

Prepared according to general procedure A. 3-Benzylcyclohex-2-en-1-one (177 mg, 0.95 mmol, 1.0 equiv.), $CeCl_3$ (234 mg, 0.95 mmol, 1.0 equiv.) and $NaBH_4$ (72 mg, 1.9 mmol, 2.0 equiv.) in MeOH (5 mL) gave the titled compound (134 mg, 0.71 mmol, 75%), which required no further purification.

<u>*NMR*</u>
^{1}H NMR (400 MHz, $CDCl_3$) δ 7.37 – 7.26 (3H, m, ArH), 7.23 – 7.13 (2H, m, ArH), 5.54 (1H, dt, *J* = 3.3, 1.6 Hz, CH), 4.26 – 4.15 (1H, m, CH), 3.28 (2H, s, CH_2Ph), 1.98 – 1.64 (4H, m, CH_2), 1.61 – 1.47 (2H, m, CH_2); ^{13}C NMR (101 MHz, $CDCl_3$) δ 141.6 (C), 139.5 (C), 129.0 (ArCH), 128.4 (2 x ArCH), 126.2 (2 x ArCH), 125.9 (CH), 66.1 (CH), 44.3 (CH_2Ph), 31.9 (CH_2), 28.3 (CH_2), 19.2 (CH_2).

Data consistent with literature.[5]

2.2 Enals and Enones

The following compounds are commercially available: cyclohexenone, trans-2-methyl-2-butenal, hexananal, cyclohex-1-enecarbaldehyde, cinnamaldehyde and citral.

The following compounds were synthesised as stated in a previous publication.[2]

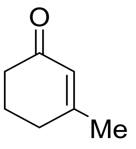

2a

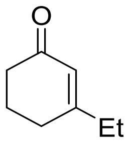

2b

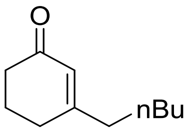

2c

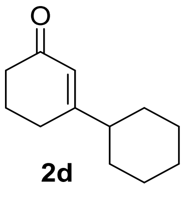

2d

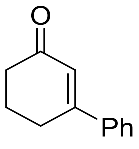

2e

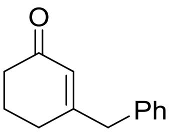

2f

2.3 Aldehydes and Ketones

The following compounds are commercially available: cyclohexanone, 3-methylcyclohexanone, 2-methylbutyraldehyde, cyclohexanecarboxaldehyde, 3-phenylpropanal, citronellal.

The following compounds were synthesised as stated in our previous publication.[2]

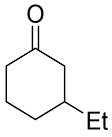

4b

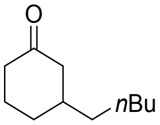

4c

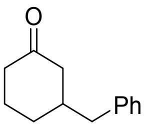

4f

3-Cyclohexylcyclohexanone (4d)

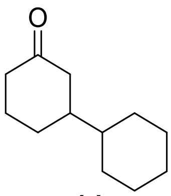

4d

Prepared according to *General Procedure C*. 3-Cyclohexylcyclohex-2-enone (35 mg, 0.20 mmol, 1.0 equiv.) 10% Pd/C (5 mg, 0.05 mmol, 25 mol%) in EtOAc (1 mL). Purification over silica gel (10% EtOAc in petroleum ether) gave the titled compound (12 mg, 0.07 mmol, 33%) as a colourless oil.

<u>*NMR*</u>

^{1}H NMR (400 MHz, $CDCl_3$) δ 2.41 – 2.30 (2H, m, CH_2), 2.28 – 2.18 (1H, m, CH_2), 2.13 – 2.01 (2H, m, CH_2), 1.91 – 1.82 (1H, m, CH_2), 1.78 – 1.52 (7H, m, CH_2, CH), 1.44 – 1.31 (1H, m, CH_2), 1.28 – 1.06 (4H, m, CH_2, CH), 1.03 – 0.90 (2H, m, CH_2); ^{13}C NMR (101 MHz, $CDCl_3$) δ 212.9 (C), 45.7 (CH_2), 44.8 (CH), 42.8 (CH), 41.8 (CH_2), 30.1 (CH_2), 30.0 (CH_2), 28.6 (CH_2), 26.7 (CH_2), 26.7 (CH_2), 26.7 (CH_2), 25.8 (CH_2).

Data consistent with literature.[6]

3-Phenylcyclohexanone (4e)

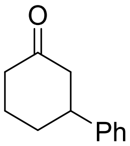

4e

Prepared using *General Procedure C:* Enone (173 mg, 1.0 mmol) and 10% Pd/C (9 mg, 0.09mmol, 9 mol%) in EtOAc (2 mL) gave the crude product. Purification by column chromatography (30% EtOAc/petroleum ether) (Rf = 0.49) gave the titled compound (59 mg, 0.34mmol, 34%) as a pale-yellow oil.

^{1}H NMR (400 MHz, $CDCl_3$) δ 7.30 – 7.25 (2H, m, ArH), 7.21 – 7.15 (3H, m, ArH), 2.96 (1H, tt, *J* = 11.7, 4.0 Hz, CH), 2.58 – 2.51 (1H, m, CH_2), 2.49 (1H, dd, *J* = 12.2, 1.1 Hz, CH_2), 2.45 – 2.37 (1H, m, CH_2), 2.37 – 2.27 (1H, m, CH_2), 2.14 – 1.99 (2H, m, CH_2), 1.86 – 1.66 (2H, m, CH_2).

^{13}C NMR (101 MHz, $CDCl_3$) δ 211.1 (C), 144.4 (C), 128.8 (ArH), 126.8 (ArH) , 126.7 (ArH), 49.0 (CH_2), 44.8 (CH), 41.3 (CH_2), 32.9 (CH_2), 25.6 (CH_2).`

Data consistent with literature.[7]

2.4 Intermediate allylic amines

The following compounds were synthesised as stated in a previous publication.[2]

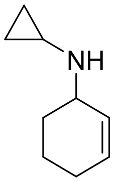

5g

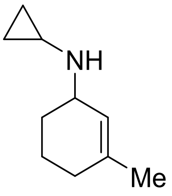

5a

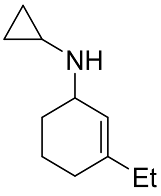

5b

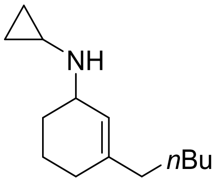

5c

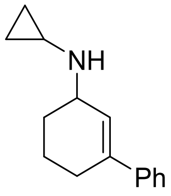

5e

N-(3,7-Dimethylocta-2,6-dien-1-yl)cyclopropanamine (5o)

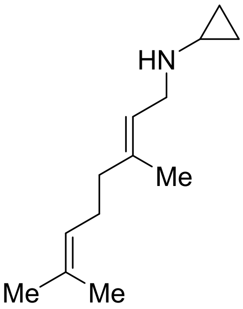

5o

Cyclopropylamine(124 µL, 2 mmol, 2 equiv.) was added to a solution of citral (mixture of *cis* and *trans*)(0.17 mL, 1 mmol, 1.0 equiv.) in EtOH (4 mL) at room temperature and stirred for 6 hours. Sodium triacetoxyborohydride (933 mg, 4.4 mmol, 4.4 equiv.) was then added portion wise. The resulting solution was left to stir at room temperature for 16 hours, before being quenched by the addition of sat. $NaHCO_3$(aq) (5 mL and an extraction into Et_2O (3 x 5 mL). The combined organics were dried ($MgSO_4$) and concentrated to give the titled compound (154 mg, 0.80 mmol, 80%) which required no further purification.

NMR

^1H NMR (400 MHz, $CDCl_3$) δ 5.34 – 5.22 (1H, m, CH), 5.15 – 5.04 (1H, m, CH), 3.27 (1H, dd, *J* = 12.4, 7.0 Hz, CH_2), 3.23 – 3.17 (1H, m, CH_2), 2.25 – 1.95 (6H, m, NH, CH_2 and CH), 1.74 – 1.56 (9H, m, CH_3), 0.50 – 0.32 (4H, m, CH_2); ^{13}C NMR (101 MHz, $CDCl_3$) δ 137.9 (C), 131.7(C), 124.3 (CH), 121.2 (CH), 51.7 (CH_2), 47.0 (CH_2), 39.8 (CH_2), 30.2 (CH), 26.7 (CH_3), 25.8 (CH_3), 17.8 (CH_2), 16.4 (CH_2), 6.4 (CH_3). Note only peaks corresponding to major isomer are shown.

Data consistent with literature.[8]

2.5 Synthesis of racemic saturated amines

The following compounds were synthesised as stated in our previous publication.[2]

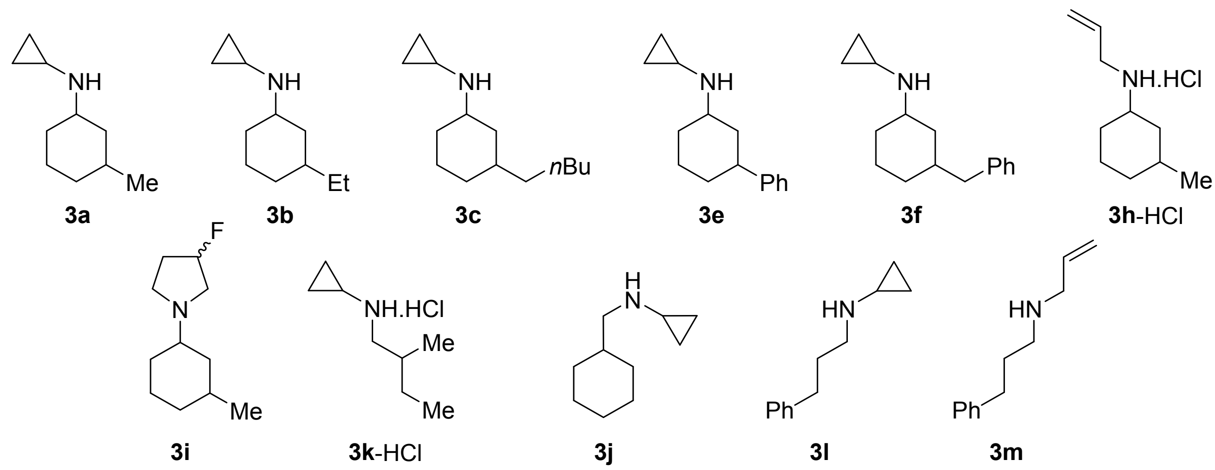

N-Cyclopropylcyclohexylamine (3g)

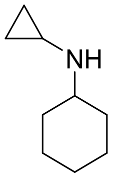

3g

Prepared according to *General Procedure B*. Cyclohexanone (0.21mL, 2.0 mmol, 1.0 equiv.), acetic acid (0.11 mL, 2.0 mmol, 1.0 equiv.) sodium triacetoxyborohydride (636 mg, 3.0 mmol, 1.5 equiv.), cyclopropylamine (0.15 mL, 2.2 mmol, 1.1 equiv.) and anhydrous THF (10 mL) gave the titled compound (43 mg, 0.31 mmol, 16%) as a pale brown oil, which required no further purification.

<u>*NMR*</u>

^{1}H NMR (400 MHz, $CDCl_3$) δ 2.59 (1H, tt, *J* = 10.6, 3.8 Hz, CH), 2.18 – 2.11 (1H, m, CH), 2.00 – 1.92 (2H, m, CH_2), 1.78 – 1.56 (4H, m, CH_2), 1.35 – 1.03 (m, 4H), 0.49 – 0.35 (4H, m, CH_2(*cyclopropyl*)); ^{13}C NMR (101 MHz, $CDCl_3$) δ 57.5 (CH) 33.7 (CH_2), 28.3 (CH), 26.2 (CH_2), 25.2 (CH_2), 6.3 (CH_2).

Data consistent with literature.[9]

rac-N-Cyclopropylcitronellamine (3o)

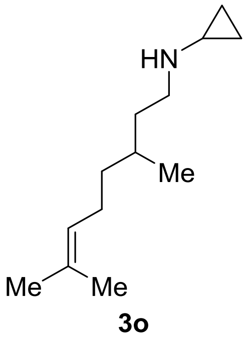

3o

Glacial acetic acid (57 µL, 1.0 mmol, 1.0 equiv.) and sodium triacetoxyborohydride (318 mg, 1.5 mmol, 1.5 equiv.) were added to a solution of citronellal (180 µL, 1.0 mmol, 1.0 equiv.), cyclopropylamine (62 µL, 1.1 mmol, 1.1 equiv.) in anhydrous THF (5 mL) under inert atmosphere. The reaction mixture was stirred at room temperature for 18 hours before the addition of aqueous $NaHCO_3$ (sat., 5 mL) and extracted with EtOAc (3 × 5 mL). The combined organics were washed with 1M HCl (3 mL), before basifying to pH 12. The resulting aqueous solution was extracted with EtOAc (3 × 5 mL), dried ($MgSO_4$) and concentrated *in vacuo* to give the titled compound (40 mg, 0.20 mmol, 20%), as a yellow oil, which required no further purification.

<u>*NMR*</u>

^{1}H NMR (400 MHz, $CDCl_3$) δ 5.09 (1H, tdt, *J* = 7.1, 2.9, 1.5 Hz, CH), 2.69 (2H, tdq, *J* = 11.6, 9.4, 5.8 Hz, CH_2), 2.11 (1H, tt, *J* = 6.6, 3.6 Hz, NCH), 2.05 – 1.90 (2H, m, CH_2), 1.68 (3H, d, *J* = 1.3 Hz, CH_3), 1.60 (3H, s, CH_3), 1.56 – 1.42 (2H, m, CH_2), 1.38 – 1.24 (2H, m, CH_2), 1.21 – 1.11 (1H, m, CH), 0.89 (3H, d, *J* = 6.6 Hz, CH_3), 0.45 – 0.39 (2H, m, Cypropyl-CH_2), 0.35 – 0.30 (2H, m, Cypropyl-CH_2); ^{13}C NMR (101 MHz, $CDCl_3$) δ 131.3 (C), 125.0 (CH), 47.7 (CH_2), 37.5 (CH_2), 37.4 (CH_2), 30.8 (CH), 30.6 (CH), 25.9 (CH_3), 25.7 (CH_2), 19.8 (CH_3), 17.8 (CH_3), 6.4 (CH_2), 6.5 (CH_2). Data consistent with literature.[10]

rac-N-Cyclopropyl[3-bi(cyclohexyl)yl]amine (3d)

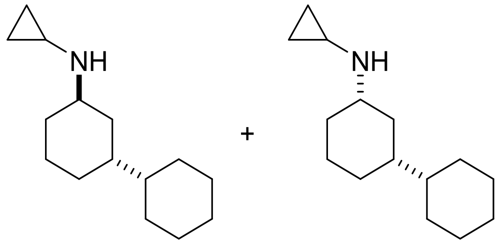

3d

Experimental Procedure C. 3-Cyclohexylcyclohex-2-enone (60 mg, 0.34 mmol, 1.0 equiv.), Pd/C (5 mg, 0.05 mmol, 15 mol%) in EtOAc (3 mL) gave the crude product which was taken onto the next step with no further purification.

Experimental Procedure B. Crude 3-Cyclohexylcyclohexanone (0.34 mmol), cyclopropylamine (30 μL, 0.37 mmol, 1.1 equiv.), sodium triacetoxyborohydride (108 mg, 0.51 mmol, 1.5 equiv.), acetic acid (19 μL, 0.34 mmol, 1.0 equiv.) in anhydrous THF (3mL) gave the title compound as an inseparable mixture of diastereoisomers (19 mg, 0.09 mmol, 25%, dr 27:73).

NMR

^{1}H NMR (400 MHz, $CDCl_3$) δ 3.01 – 2.91 (1H, m, CH), 2.08 (1H, app. tt, *J* = 6.6, 3.7 Hz, CH), 1.77 – 1.52 (8H, m, CH_2), 1.51 – 1.44 (3H, m, CH_2 and CH), 1.39 – 1.32 (2H, m, 1H from CH_2 and CH), 1.27 – 1.05 (5H, m, CH_2), 1.00 – 0.84 (2H, m, CH_2), 0.46 – 0.39 (2H, m, CH_2(*cyclopropyl*)), 0.34 – 0.29 (2H, m, CH_2(*cyclopropyl*)); ^{13}C NMR (101 MHz, $CDCl_3$) δ 52.9 (CH), 37.2 (CH), 34.3 (CH_2), 31.5 (CH_2), 30.4 (CH), 29.4 (CH_2), 28.5 (CH), 26.9 (CH_2), 26.8 (CH_2), 20.8 (CH_2), 6.3 (CH_2), 6.2 (CH_2). Note: data assignment for major diastereoisomer.

Data consistent with literature.[2]

Enantiomerically Enriched Products

Enantiomerically enriched samples of **3a** and **3i** was prepared using *Experimental Procedure B*. Where (*R*)-3-methylcyclohexanone was utilised in this reaction with cyclopropylamine and allyl amine respectively to give a mixture of the (1R,3R) and (1S,3R) where the (1R, 3R) is the observed major isomer.

Chiral GC traces:

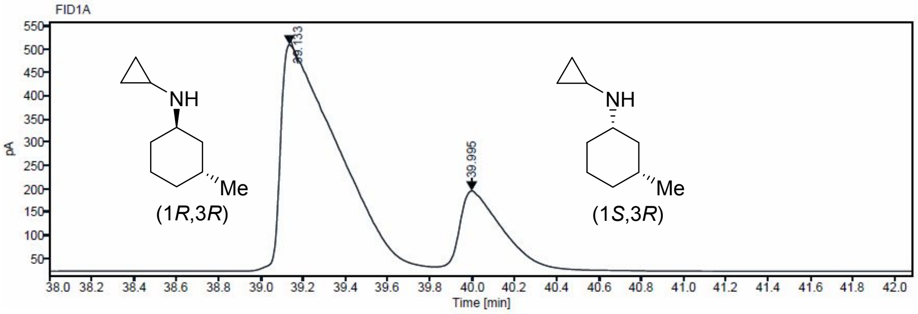

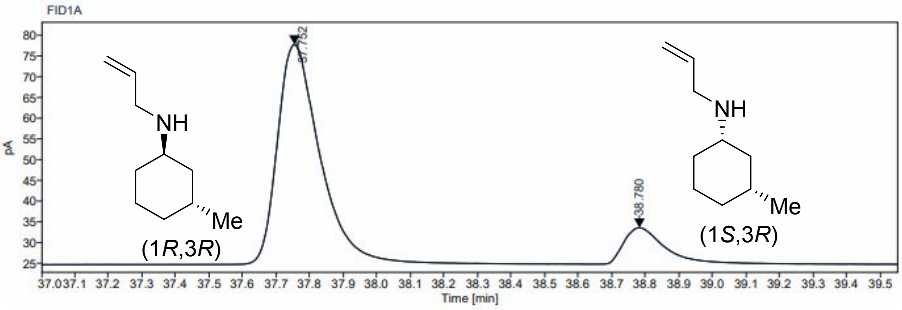

FID1A
pA
Time [min]
37.752
38.780
NH
Me
(1*R*,3*R*)
NH
Me
(1*S*,3*R*)

3.0 Biotransformations

3.1 General procedure for analytical scale biotransformations for the ShCOa oxidations (500 µL total volume)

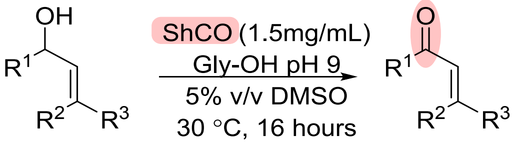

Unless stated otherwise the following components were added to a 1.5 mL Eppendorf: DMSO (20 uL, final concentration = 5% v/v), ShCO (stocks concentrations range between 7 – 12 mg/mL in 100 mM phosphate buffer pH 7.4, final concentration = 1.5 mg/mL) and *allylic alcohol* (stock concentration = 1M in DMSO, final concentration = 100 mM) and glycine buffer pH 9 (stock concentration = 100 mM to make up reactions to a final volume of 500 uL). Note that the allylic alcohol was added last to begin the reaction before the reaction mixture was shaken at 200 rpm and 30 °C for 16 hours.

The reaction mixture was extracted with EtOAc (2 x reaction volume) followed by centrifugations and the organic extractions were used directly for GC-FID and/or GCMS analysis.

3.2 Overview of allylic alcohols screened with ShCO

ShCO only reaction

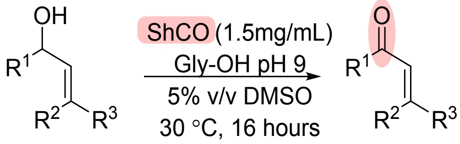

Substrates accepted by the enzyme that gave good-excellent conversions and could be isolated

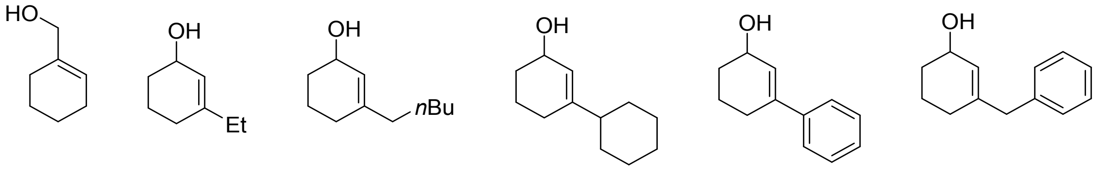

Substrates that were accepted by the enzyme, but the products either degraded in the reaction mixture or could not be isolated

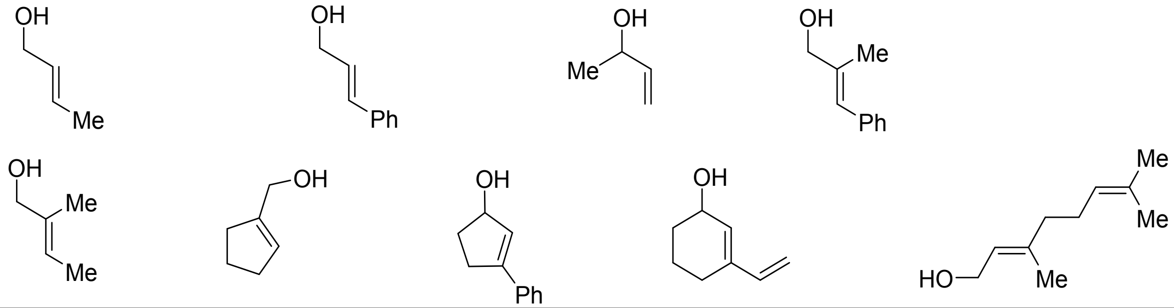

Substrates that were not accepted by the enzyme

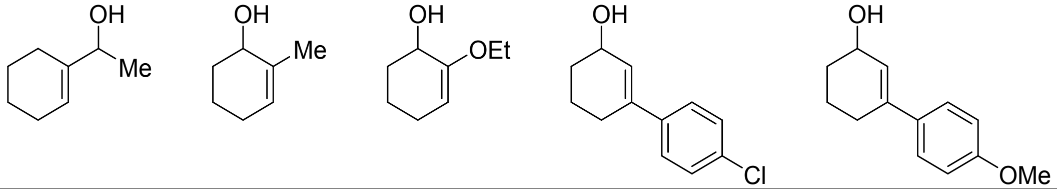

3.3 General procedure for analytical scale biotransformations for the ShCO – EneIRED cascade (500 µL total volume)

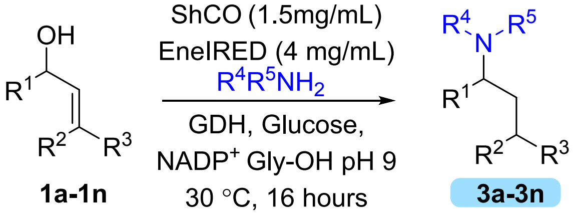

Unless stated otherwise the following components were added to a 1.5 mL Eppendorf: DMSO (20 uL, final concentration = 5% v/v), NADP+ (stock concentration = 10mg/mL in 100 mM phosphate buffer pH 7.4, final concentration = 1mg/mL), *D*-glucose (stock concentration = 100 mg/mL in dH_2O, final concentration = 10 mg/mL), GDH (stock concentration = 10 mg/mL in 100 mM phosphate buffer pH 7.4), EneIRED (stock concentration = 40 mg/mL in 100 mM phosphate buffer pH 7.4, final concentration 4 mg/mL), *amine* (stock concentration = 500 mM in glycine buffer pH 9, final concentration = 200 mM with pH corrected to 9), ShCO (stocks concentrations range between 7 – 12 mg/mL in 100 mM phosphate buffer pH 7.4, final concentration = 1.5 mg/mL) and *allylic alcohol* (stock concentration = 1M in DMSO, final concentration = 100 mM) and glycine buffer pH 9 (stock concentration = 100 mM to make up reactions to a final volume of 500 uL). Note that the allylic alcohol was added last to begin the reaction before the reaction mixture was shaken at 200 rpm and 30 °C for 16 hours.

The reactions were ended by the addition of 5.0 M NaOH (aq. 0.1 x reaction mixture volume), clarified by centrifugation and extract into EtOAc (2 x reaction volume) followed by further centrifugation. The organic extractions were used directly for GC-FID and/or GCMS analysis.

3.4 General procedure for scale up procedures for ShCO – EneIRED cascade (0.3 mmol scale)

To a 50 mL falcon tube the following components were added: NADP+ (0.5 mM), D-glucose (3 equiv., 30 mM), amine (20 equiv., 200 mM), and glycine buffer (100 mM, pH 9.0). The resulting solution was readjusted to pH 9.0. DMSO was then added (final concentration = 5% v/v), followed by addition of solutions of GDH (stock concentration = 2 mg/mL in glycine buffer pH 9.0, final concentration = 0.1 mg/mL), EneIRED (stock concentration = 320 mM in glycine buffer pH 9.0, final concentration 20 mM), and ShCOa (stock concentration 10mg/mL in glycine buffer, final concentration = 1.5 mg/mL). Finally, allylic alcohol (10 mM) was added to the reaction mixture and the reaction volume made up to 30 mL with glycine buffer, before shaking in an incubator (48 h, 200 rpm, 30 °C). Extraction with Et_2O (3 x 10mL) with centrifugation used to separate layers effectively. The organic fractions were then washed with deionised water (10 mL), dried ($MgSO_4$) and solvent removed *in vacuo* yielding the crude product.

3.4.1 Biocatalytic scale up to form *N*-cyclopropyl-3-methylcyclohexa-1-amine (3a)

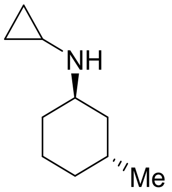

3a

Prepared according to scale-up general procedure shown in **3.4.** 3-Methylcyclohexenol (34 mg, 0.30 mmol) and cyclopropylamine (416.0 mL, 6.0 mmol) underwent oxidation followed by CR-RA in 30 mL optimised reaction medium (5% v/v DMSO in 100 mM Gly-OH pH 9.0). The product was isolated as a pale-yellow oil (19 mg, 0.12 mmol, 41%).

<u>GCMS trace from scale up reaction:</u>

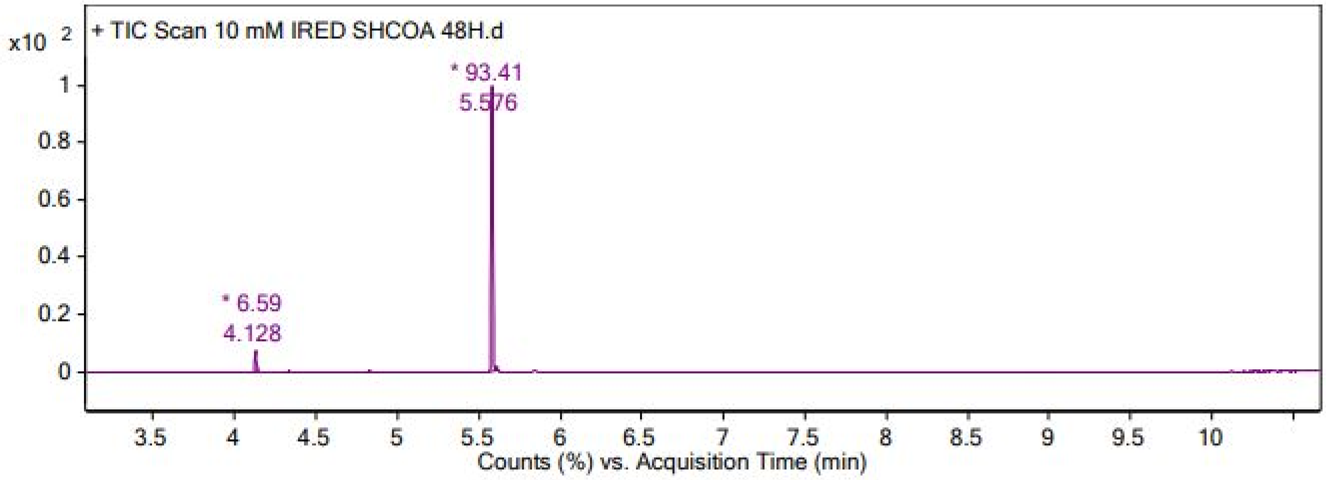

^1H NMR of isolated product:

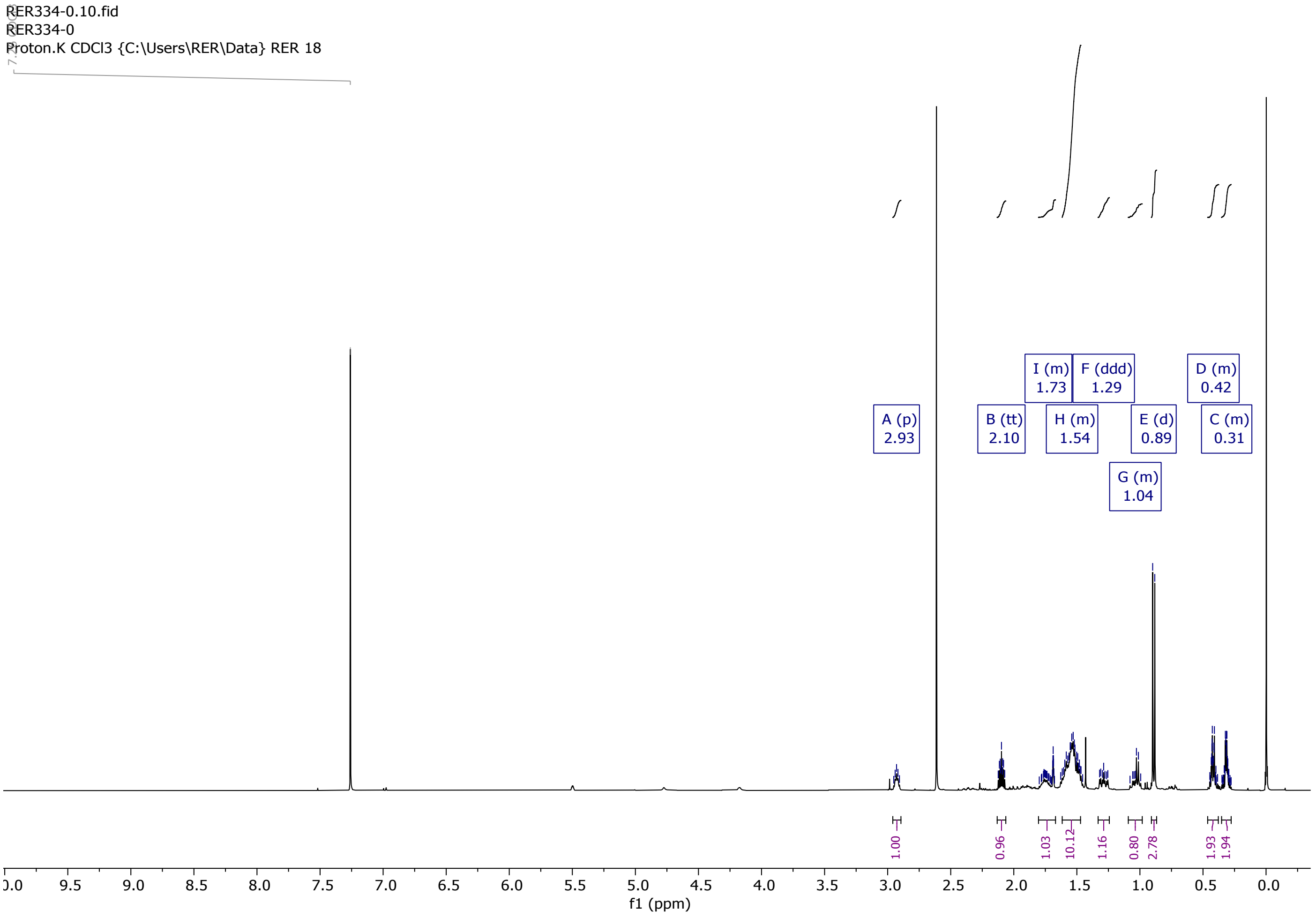

3.4.2 Biocatalytic scale up to form N-cyclopropyl-3-methylcyclohexa-1-amine (3o)

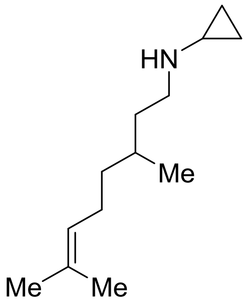

3o

Prepared according to scale-up general procedure shown in **3.4.** Nerol (46 mg, 0.30 mmol) and cyclopropylamine (416.0 mL, 6.0 mmol) underwent oxidation followed by CR-RA in 30 mL optimised reaction medium (5% v/v DMSO in 100 mM Gly-OH pH 9.0). The product was isolated as a colourless oil (26 mg, 0.13 mmol, 44%). Note: contained 10% aldimine intermediate.

^{1}H NMR of isolated product:

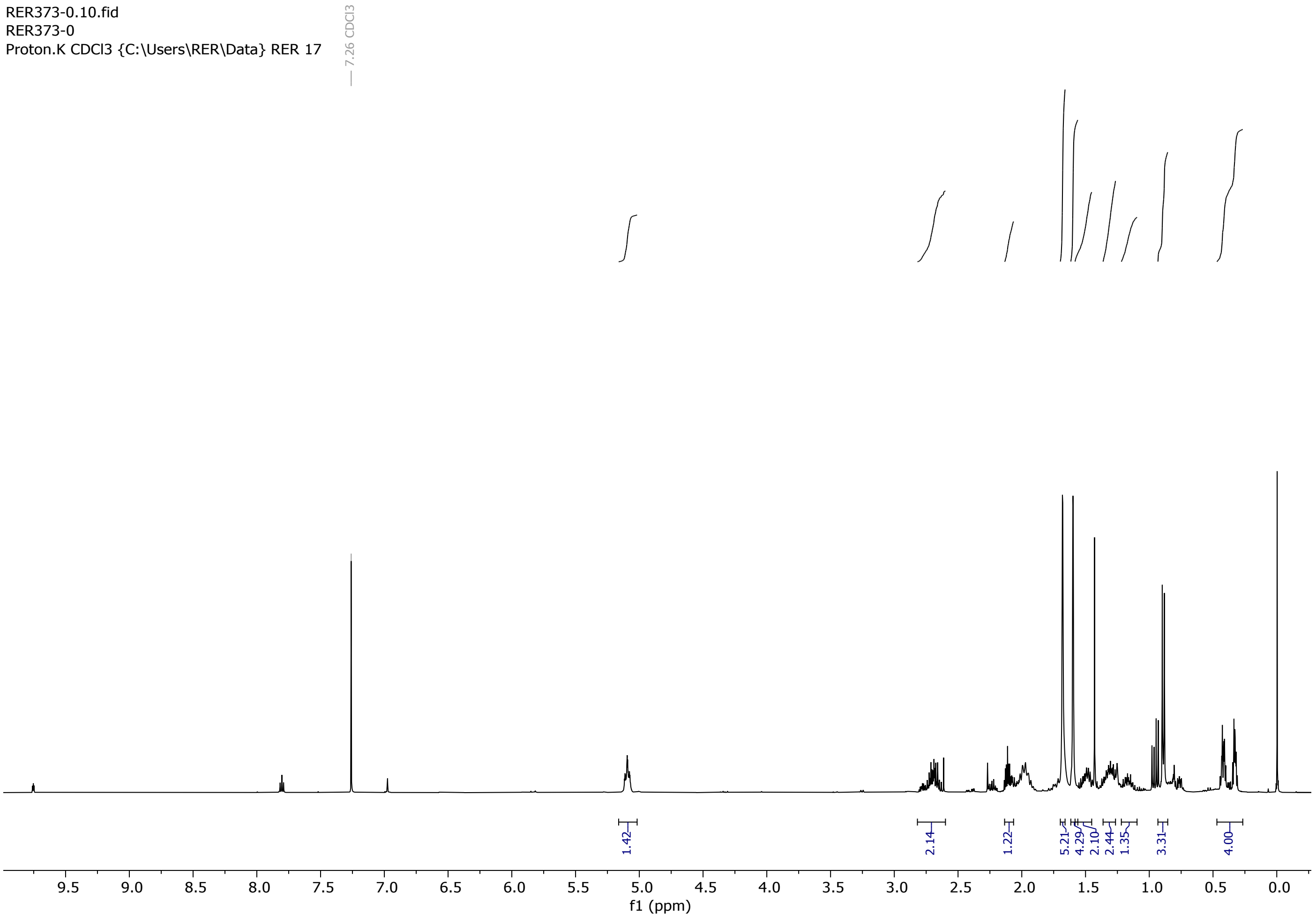

4.0 Gas Chromatography (GC) analysis of biotransformations

4.1 GC-FID Traces from optimisation of ShCOa catalysed oxidations shown in Table 1 with further examples shown.

GC-FID-rac method was used (see 1.2.1 for further details).

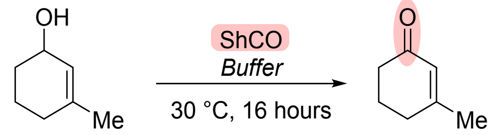

1a **2a**

Retention time of **1a** = 2.001 min.

Retention time of **2a** = 2.488 min.

Entry in Table 1	Deviation from Entry 1	Chromatogram (GC-FID)
**1**	-	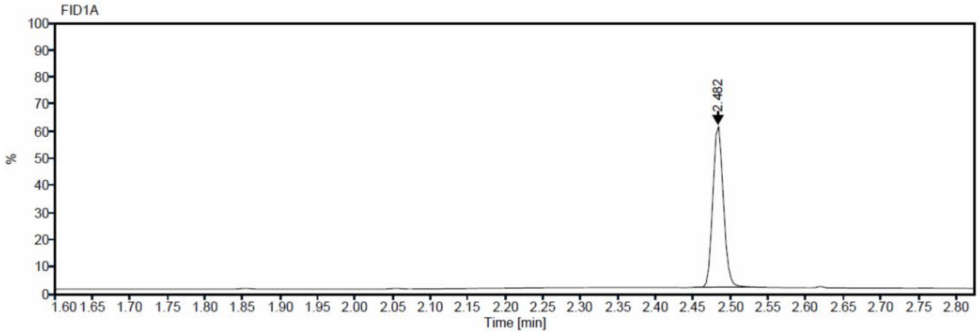<br>**Signal:** FID1A<br>**RT [min]** / **Type** / **Width [min]** / **Area** / **Height** / **Area%**<br>2.482 / MM m / 0.07 / 68.57 / 70.21 / 100.00<br>**Sum** 68.57
**-**	50 mM **1a**	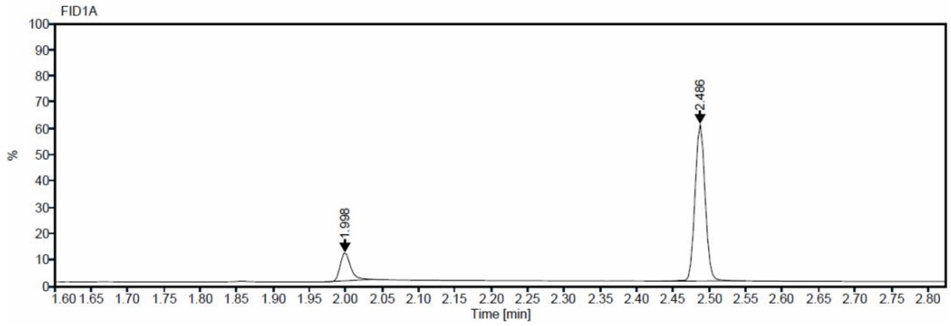<br>**Signal:** FID1A<br>**RT [min]** / **Type** / **Width [min]** / **Area** / **Height** / **Area%**<br>1.998 / MM m / 0.07 / 45.67 / 46.33 / 15.51<br>2.486 / MM m / 0.11 / 248.69 / 257.01 / 84.49<br>**Sum** 294.36
**2**	GlyOH buffer	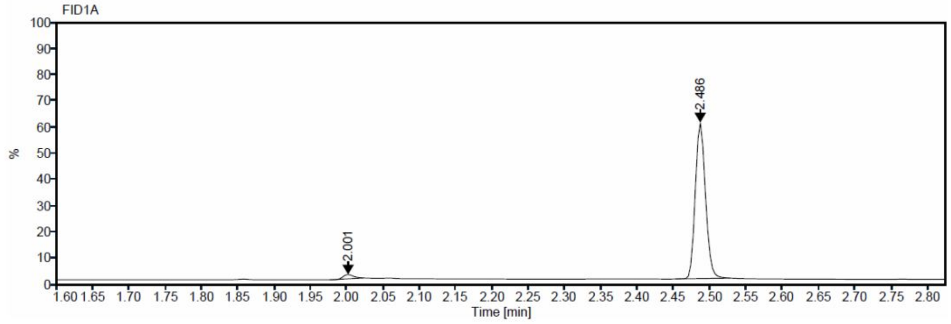

<table>
<tr><td></td><td></td><td>
Signal: FID1A
<table>
<tr><th>RT [min]</th><th>Type</th><th>Width [min]</th><th>Area</th><th>Height</th><th>Area%</th></tr>
<tr><td>2.001</td><td>MM m</td><td>0.05</td><td>2.27</td><td>2.38</td><td>2.61</td></tr>
<tr><td>2.486</td><td>MM m</td><td>0.08</td><td>84.75</td><td>86.74</td><td>97.39</td></tr>
<tr><td></td><td></td><td>Sum</td><td>87.01</td><td></td><td></td></tr>
</table>
</td></tr>
<tr><td>-</td><td>50 mM 1a in GlyOH buffer</td><td>
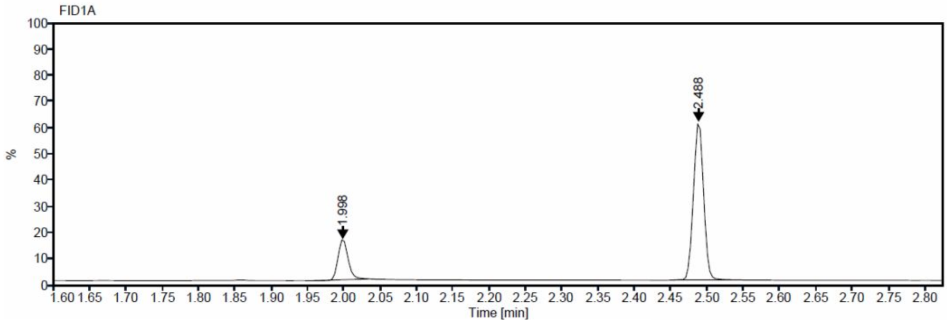

Signal: FID1A
<table>
<tr><th>RT [min]</th><th>Type</th><th>Width [min]</th><th>Area</th><th>Height</th><th>Area%</th></tr>
<tr><td>1.998</td><td>MM m</td><td>0.08</td><td>87.31</td><td>91.72</td><td>20.12</td></tr>
<tr><td>2.488</td><td>MM m</td><td>0.08</td><td>346.55</td><td>357.80</td><td>79.88</td></tr>
<tr><td></td><td></td><td>Sum</td><td>433.86</td><td></td><td></td></tr>
</table>
</td></tr>
<tr><td>3</td><td>GlyOH buffer with 5% v/v DMSO</td><td>
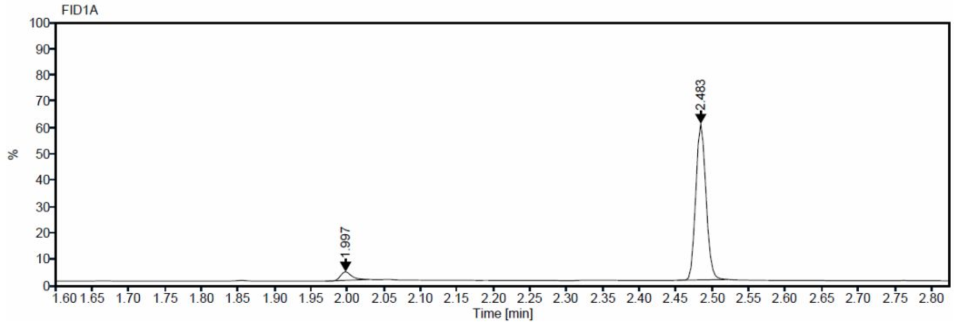

Signal: FID1A
<table>
<tr><th>RT [min]</th><th>Type</th><th>Width [min]</th><th>Area</th><th>Height</th><th>Area%</th></tr>
<tr><td>1.997</td><td>MM m</td><td>0.06</td><td>4.76</td><td>4.58</td><td>5.58</td></tr>
<tr><td>2.483</td><td>MM m</td><td>0.07</td><td>80.58</td><td>82.64</td><td>94.42</td></tr>
<tr><td></td><td></td><td>Sum</td><td>85.35</td><td></td><td></td></tr>
</table>
</td></tr>
<tr><td>-</td><td>50 mM 1a in GlyOH buffer with 5% v/v DMSO</td><td>
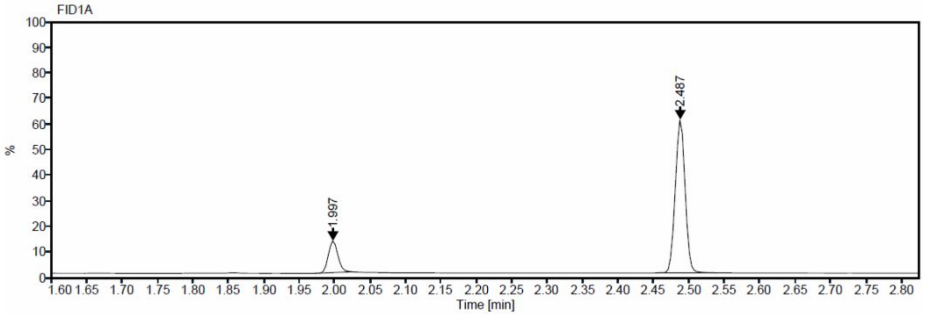

Signal: FID1A
<table>
<tr><th>RT [min]</th><th>Type</th><th>Width [min]</th><th>Area</th><th>Height</th><th>Area%</th></tr>
<tr><td>1.997</td><td>MM m</td><td>0.05</td><td>74.55</td><td>79.91</td><td>16.69</td></tr>
<tr><td>2.487</td><td>MM m</td><td>0.08</td><td>372.26</td><td>386.06</td><td>83.31</td></tr>
<tr><td></td><td></td><td>Sum</td><td>446.81</td><td></td><td></td></tr>
</table>
</td></tr>
<tr><td>-</td><td>GlyOH buffer using 2.0 mg/mL of ShCO</td><td>
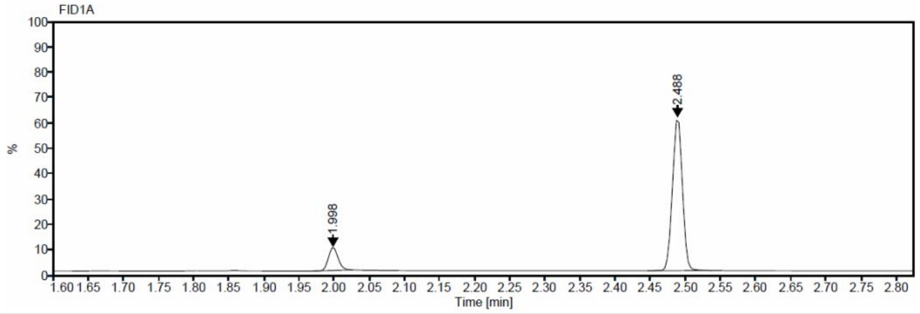

</td></tr>
</table>

<table>
<tr>
<td></td>
<td></td>
<td>
Signal: FID1A
<table>
<tr><th>RT [min]</th><th>Type</th><th>Width [min]</th><th>Area</th><th>Height</th><th>Area%</th></tr>
<tr><td>1.998</td><td>MM m</td><td>0.06</td><td>60.80</td><td>64.06</td><td>12.79</td></tr>
<tr><td>2.488</td><td>MM m</td><td>0.09</td><td>414.76</td><td>421.39</td><td>87.21</td></tr>
<tr><td></td><td></td><td>Sum</td><td>475.56</td><td></td><td></td></tr>
</table>
</td>
</tr>
<tr>
<td>-</td>
<td>50 mM 1a in GlyOH buffer using 2.0 mg/mL ShCO</td>
<td>
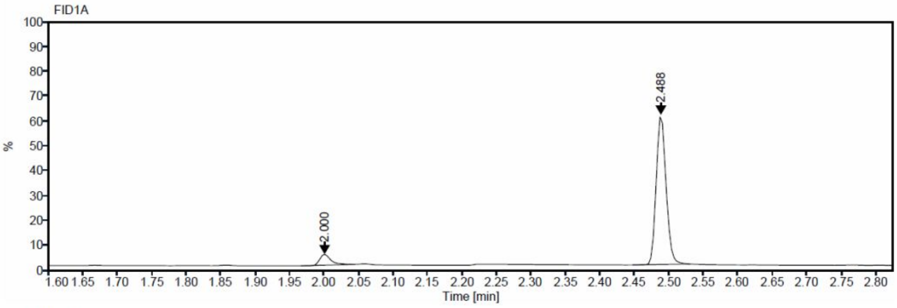

Signal: FID1A
<table>
<tr><th>RT [min]</th><th>Type</th><th>Width [min]</th><th>Area</th><th>Height</th><th>Area%</th></tr>
<tr><td>2.000</td><td>MM m</td><td>0.08</td><td>3.37</td><td>3.08</td><td>7.45</td></tr>
<tr><td>2.488</td><td>MM m</td><td>0.08</td><td>41.89</td><td>42.21</td><td>92.55</td></tr>
<tr><td></td><td></td><td>Sum</td><td>45.26</td><td></td><td></td></tr>
</table>
</td>
</tr>
</table>

4.2 GCMS/GC Traces from biotransformations shown in Scheme 2 (Oxidation only)

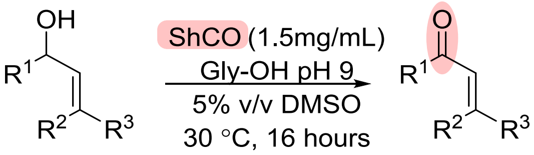

1c-1g **2c-2g**

Allylic Alcohol Starting Material	Chromatogram	Method
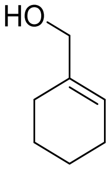 **1h** Retention time for **1h** = 4.804 min	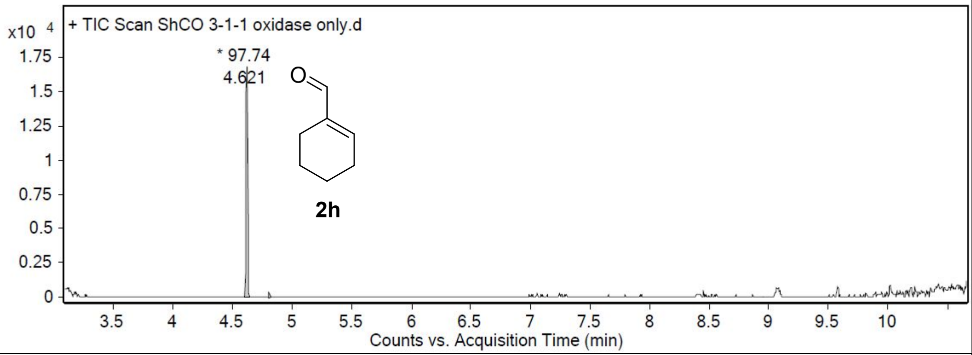	GCMS-A
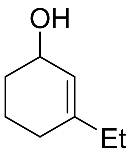 **1c**, 88% **1c** = 5.061	GCMS analysis: 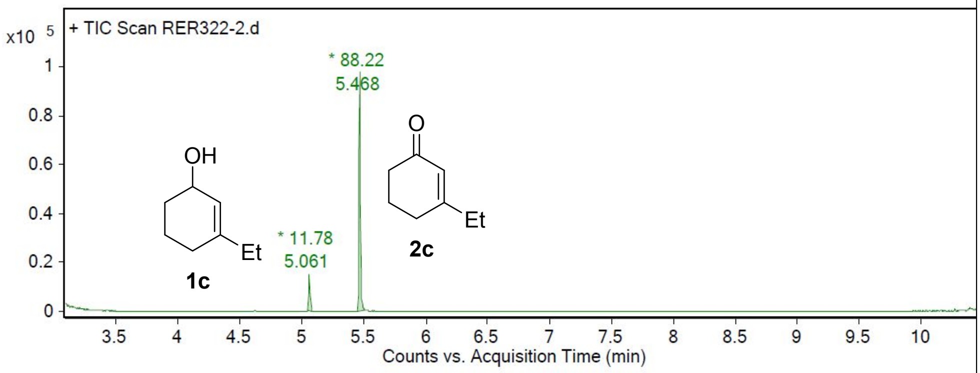	GCMS-B
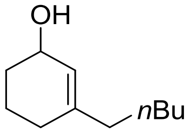 **1d** Retention time for **1d** = 6.572 min	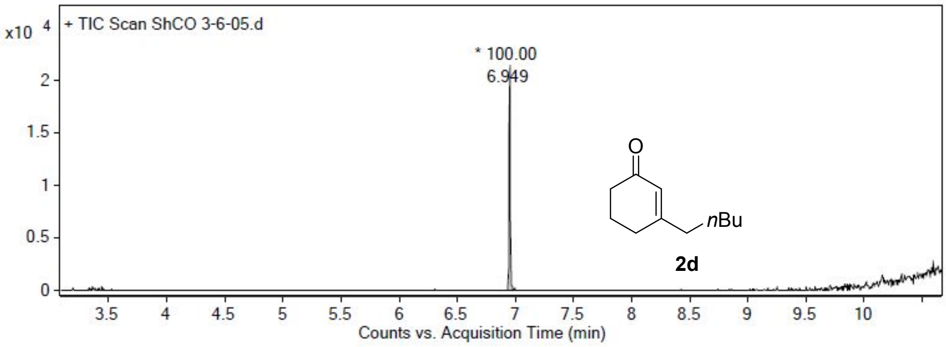	GCMS-A
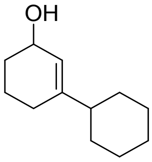 **1e** Retention time for **1e** = 7.235 min	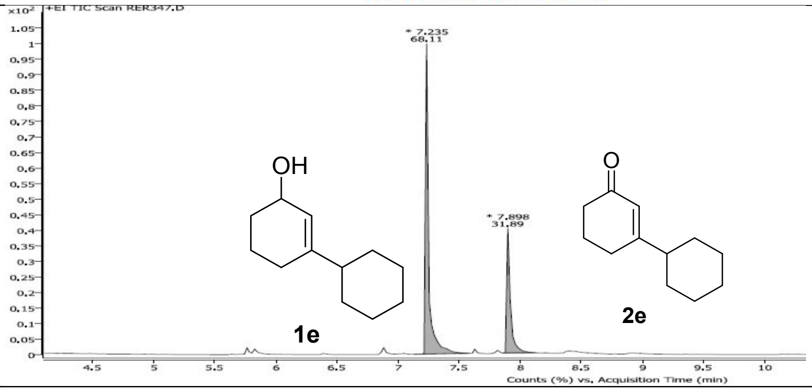	GCMS-B

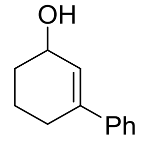<br>**1f**<br>Retention time for **1f** = 7.379 min	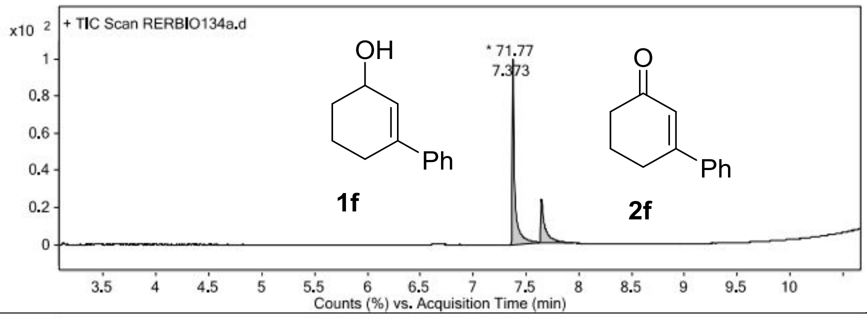	GCMS-A<br>ShCOa
	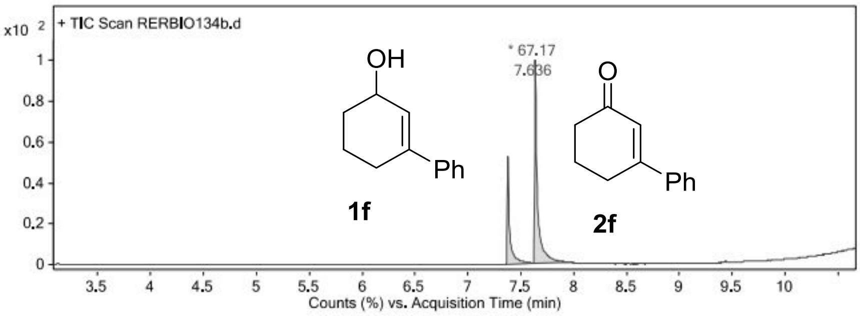	GCMS-A<br>ShCOb
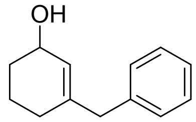<br>**1g**<br>Retention time for **1g** = 7.659 min	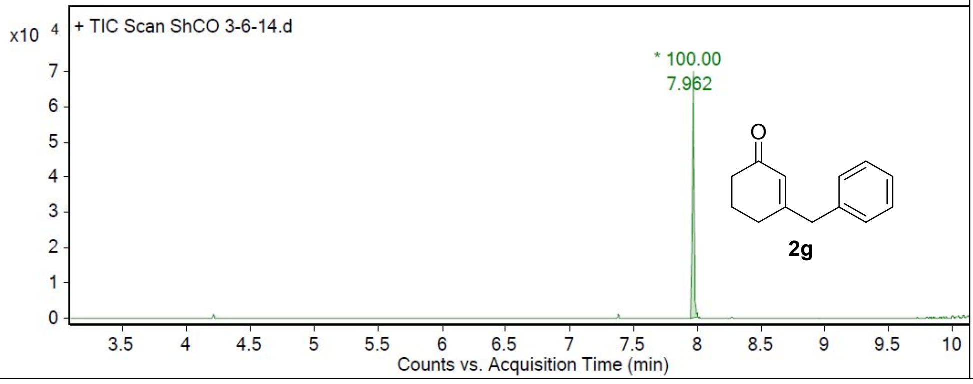	GCMS-A

4.2 GCMS Traces from biotransformations (full cascade) shown in Scheme 3

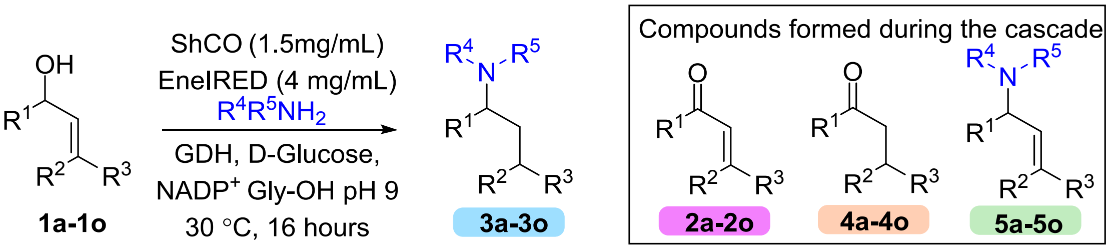

1a-1m	Amine	Chromatogram
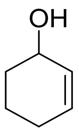 **1b**	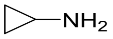	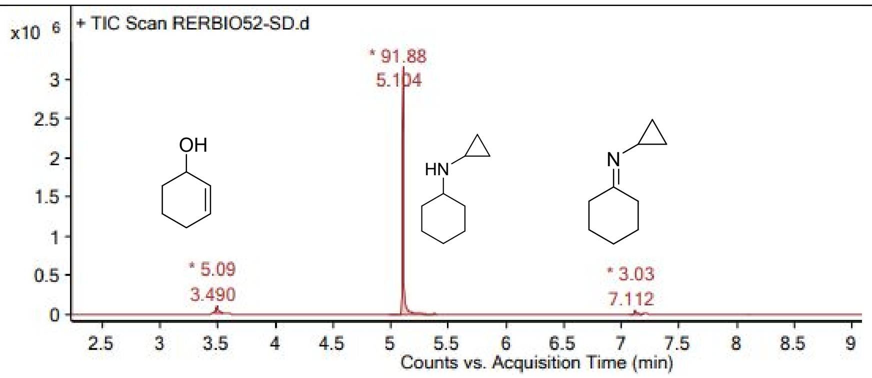 Method = GCMS-A
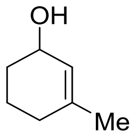 **1a**	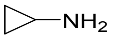	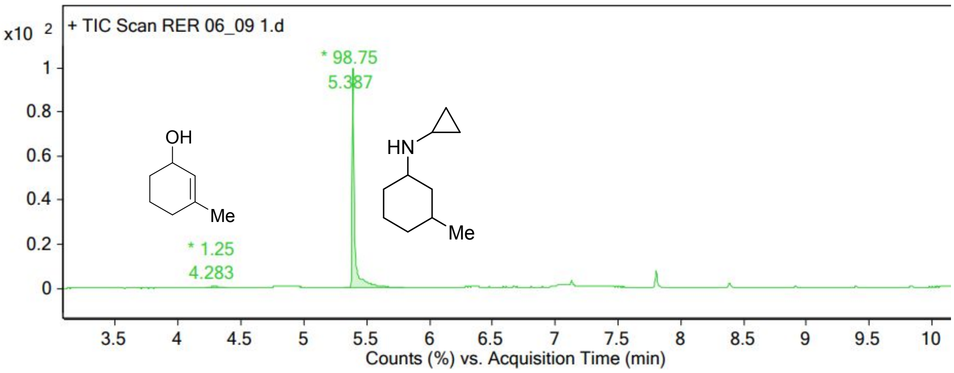 Method = GCMS-A
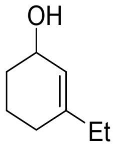 **1c**	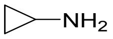	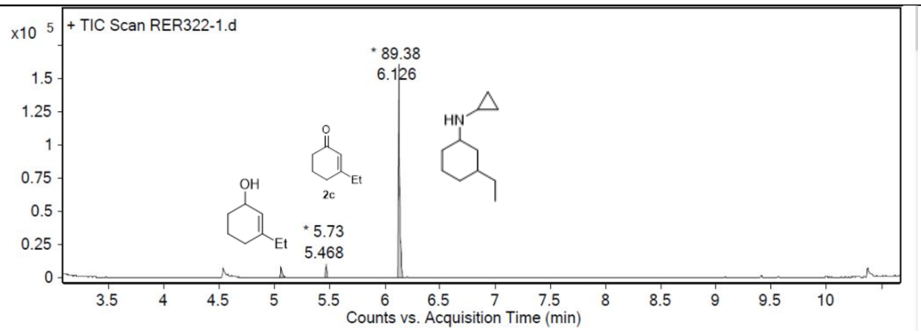 Method = GCMS-B
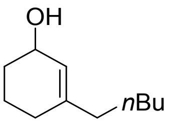 **1d**	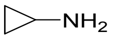	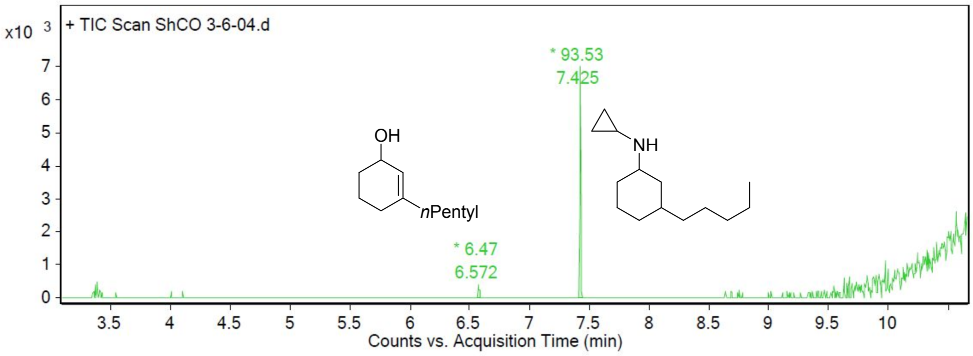 Method = GCMS-A

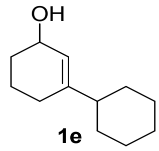<br>**1e**	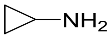	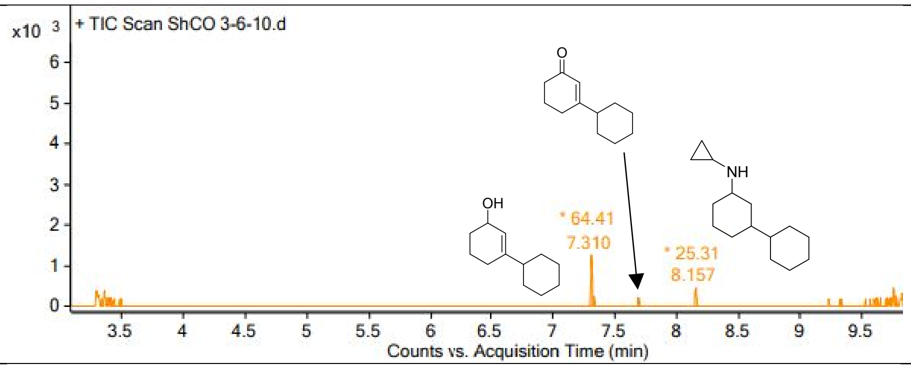
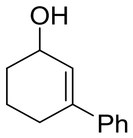<br>**1f**	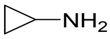	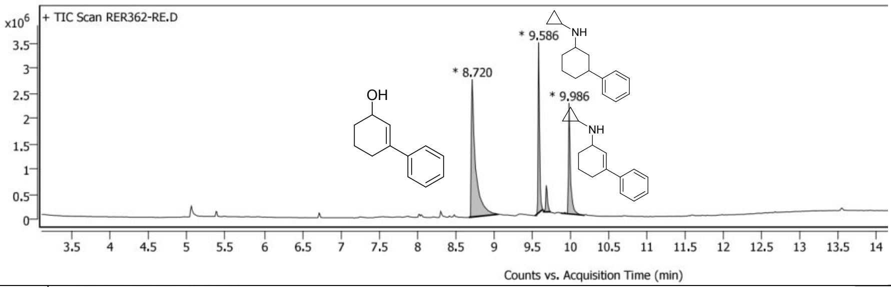
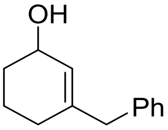<br>**1g**	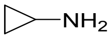	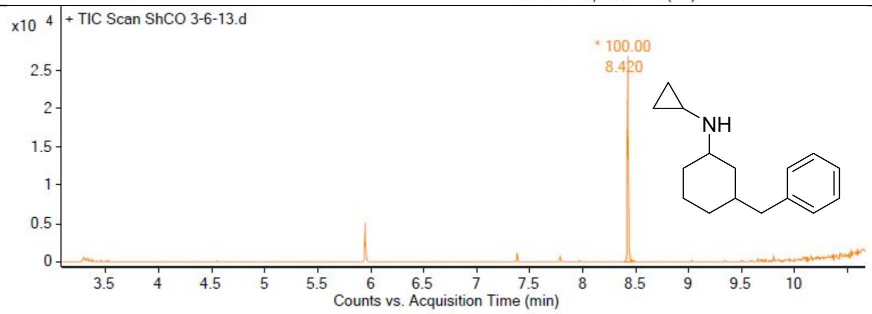
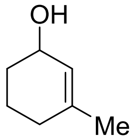<br>**1a**	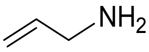	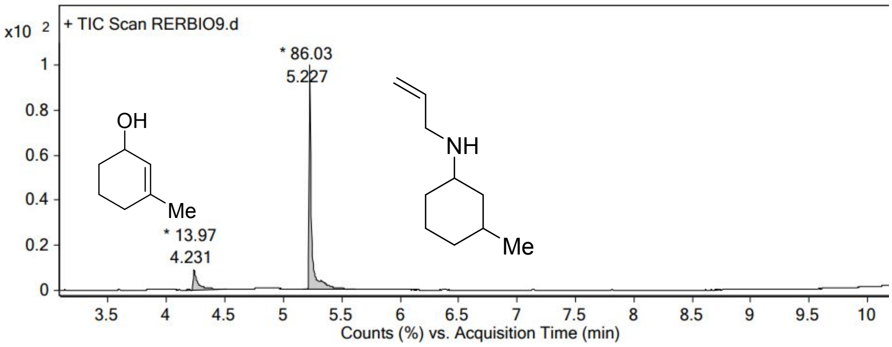
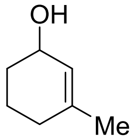<br>**1a**	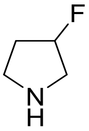	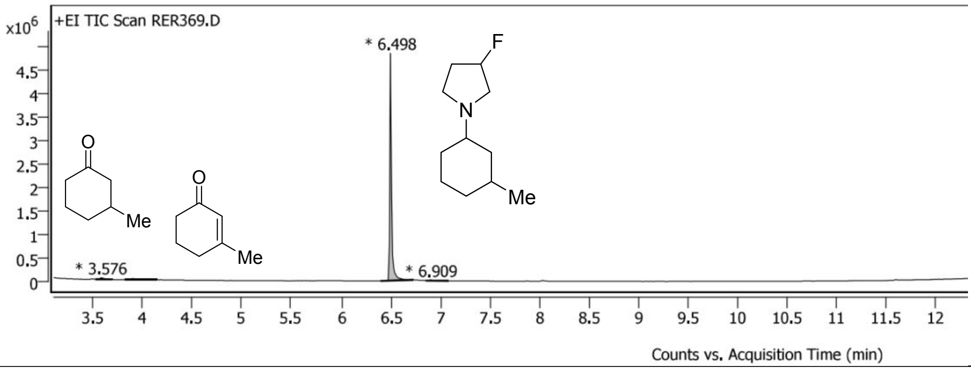

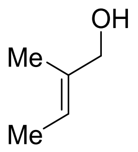<br>**1k**	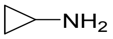	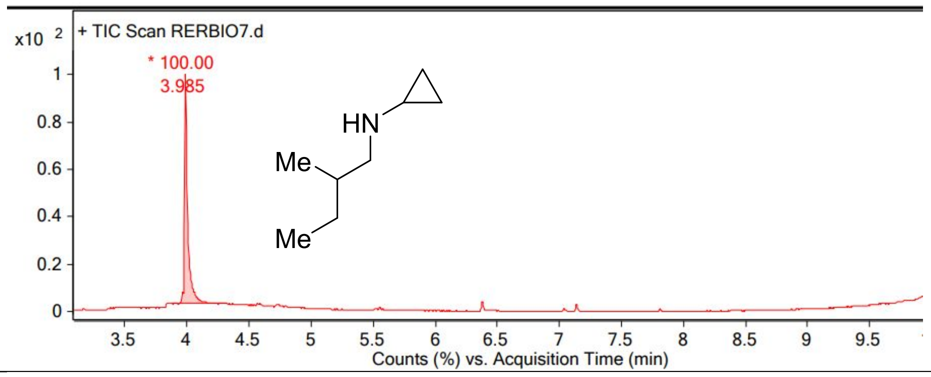
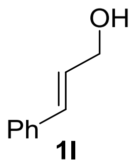<br>**1l**	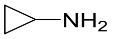	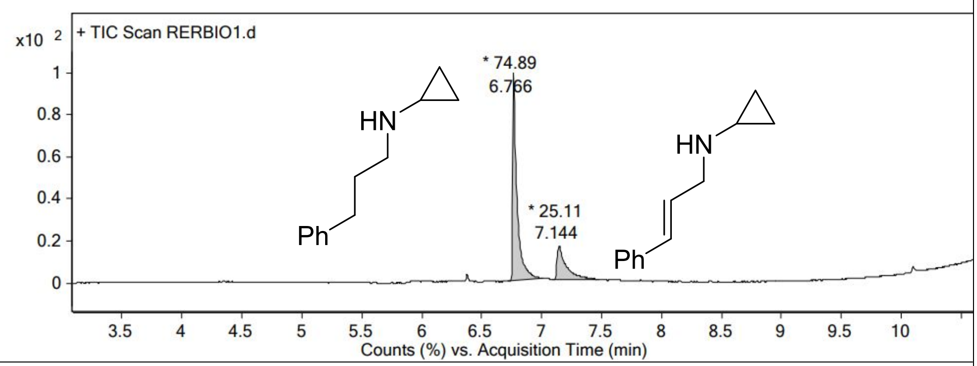
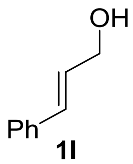<br>**1l**	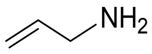	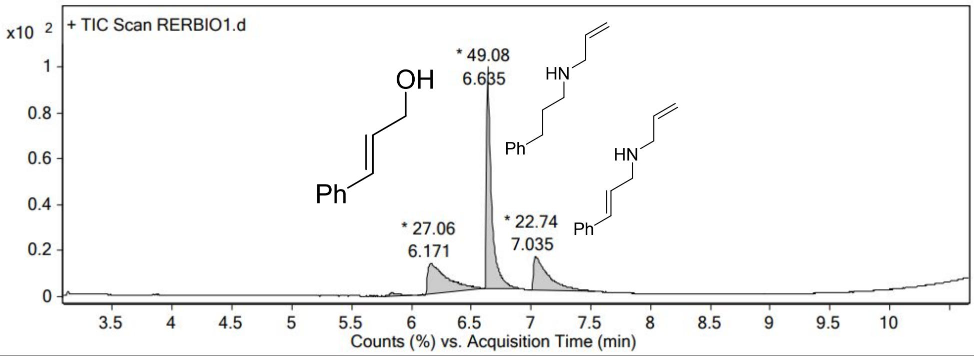
Geraniol **1n'** *(not shown in scheme 3)*<br>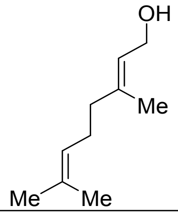	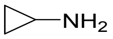	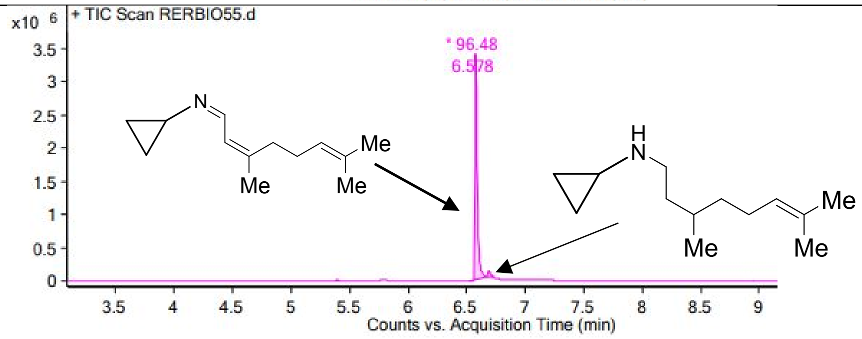
Nerol **1n**<br>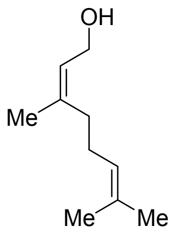	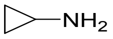	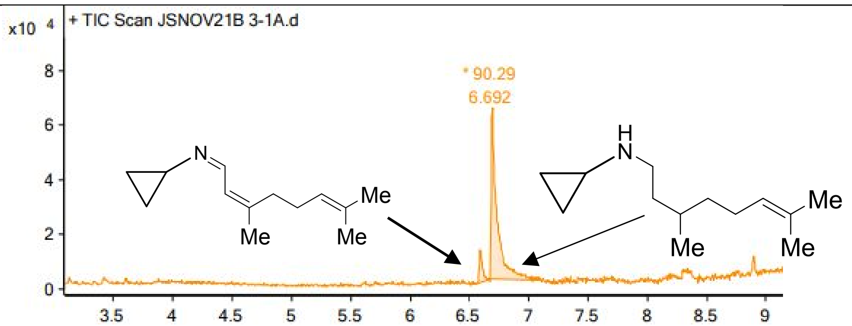

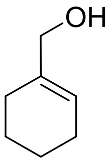<br>**1h**	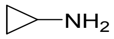	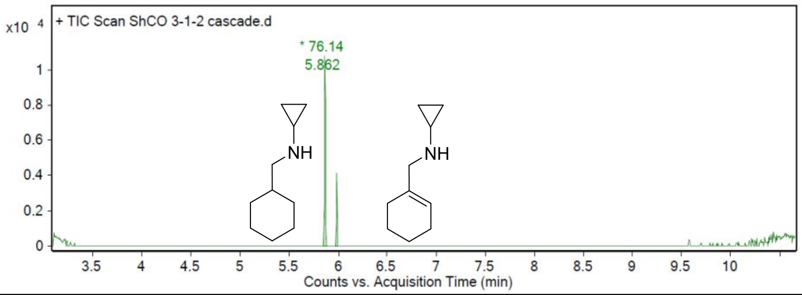x10 4<br>+ TIC Scan ShCO 3-1-2 cascade.d<br>* 76.14<br>5.862<br>1<br>0.8<br>0.6<br>0.4<br>0.2<br>0<br>3.5 4 4.5 5 5.5 6 6.5 7 7.5 8 8.5 9 9.5 10<br>Counts vs. Acquisition Time (min)<br>NH<br>NH

4.3 Chiral GC analysis

Product	Chromatograms	ee	d:r
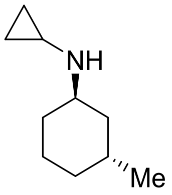 **3a**<br>*TFAA derivative*	*Racemic analytical standard:*<br>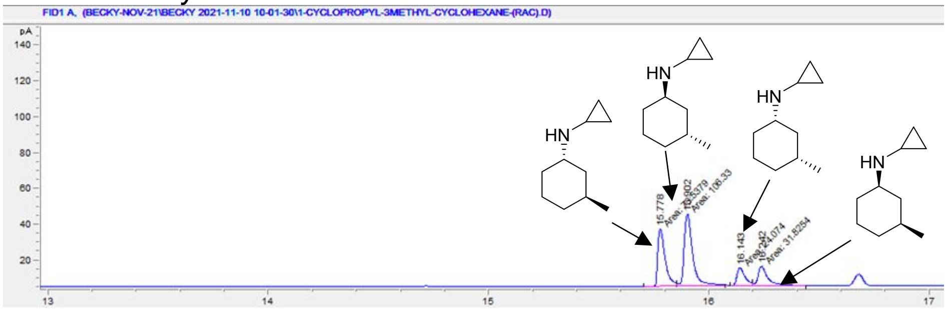<br>*Enantiomerically enriched analytical standard:*<br>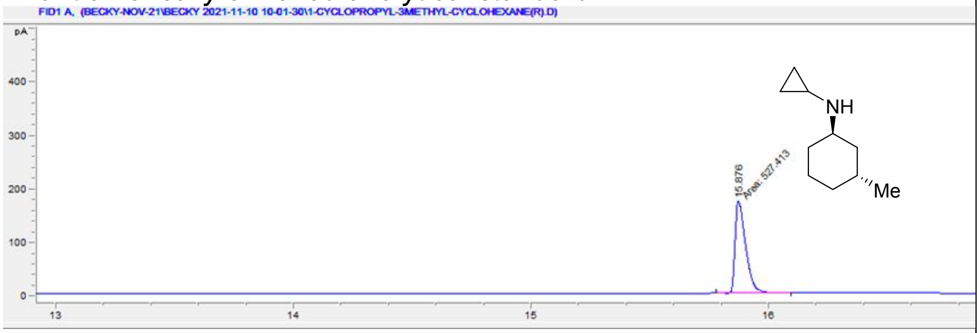<br>*Biotransformation:*<br>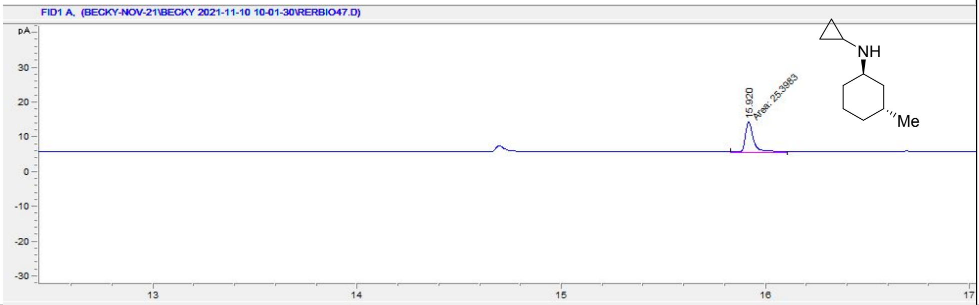	>99%	>99:1

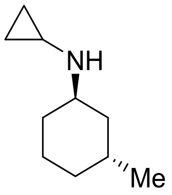<br>**3a**<br>*Free amine*	*Racemic analytical standard:*<br>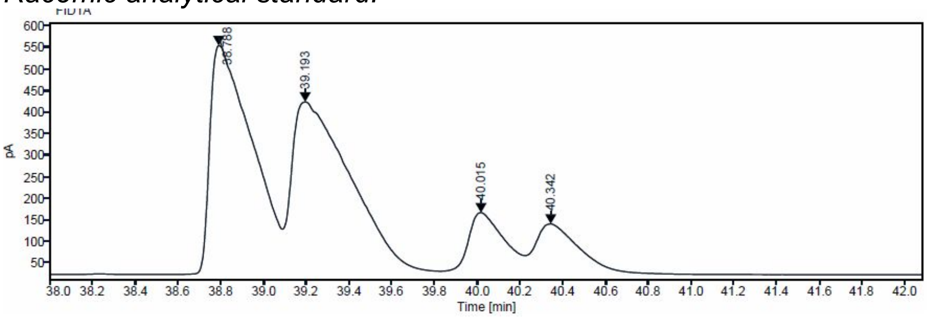<br>*Enantiomerically enriched analytical standard:*<br>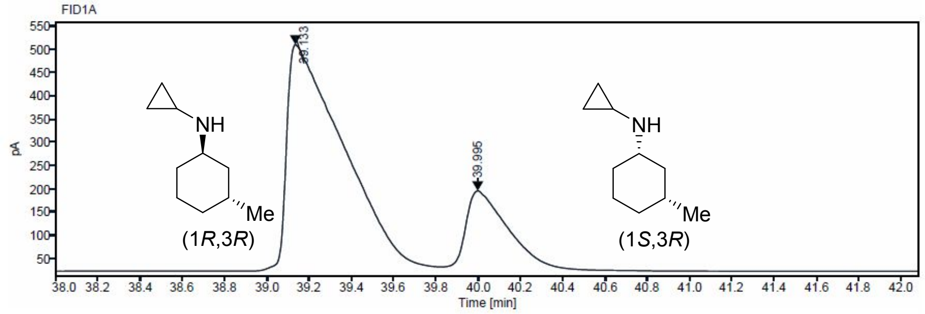<br>*Biotransformation:*<br>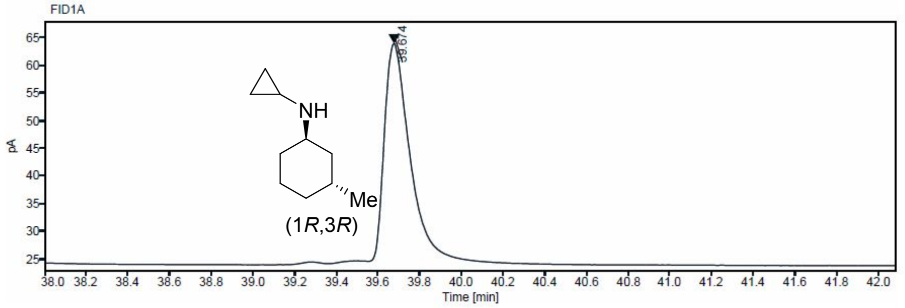<br>*Biotransformation spiked with enantiomerically enriched analytical standard:*<br>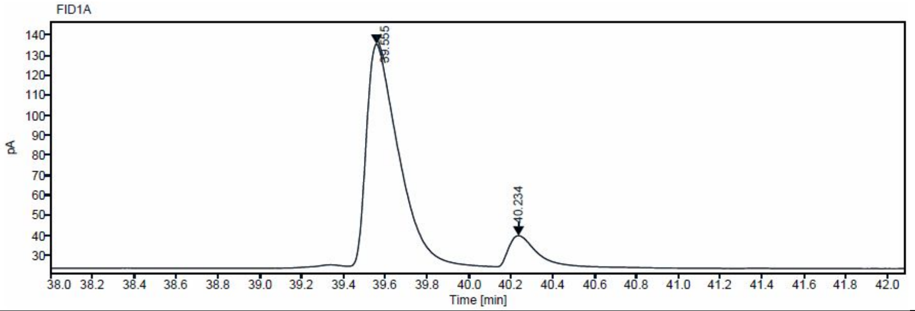	>99 %	>99:1
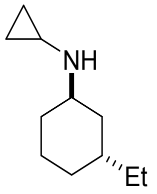<br>**3b**<br>*Free amine*	*Racemic analytical standard:*<br>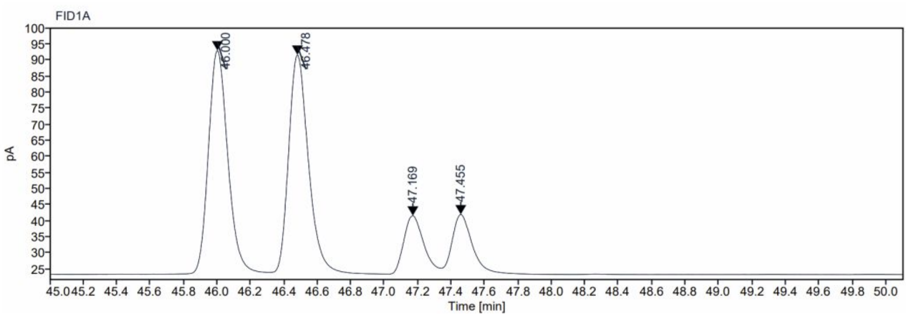<br>*Biotransformation:*	99%	98:2

<table>
<tr>
<td></td>
<td>
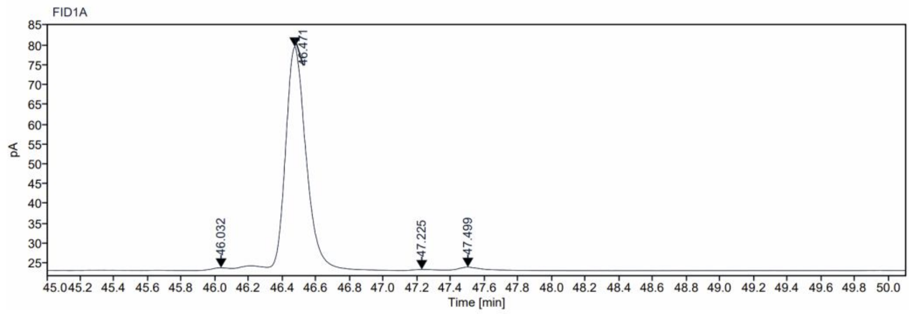

Biotransformation spiked with racemic standard:
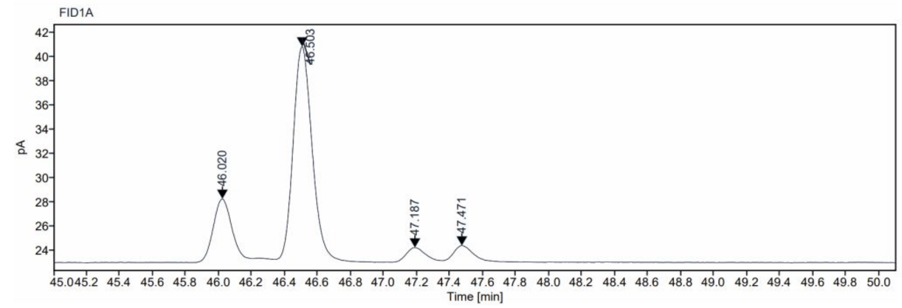

</td>
<td></td>
<td></td>
</tr>
<tr>
<td>
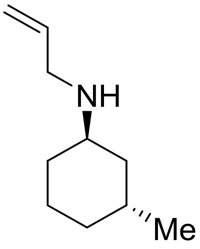

3h
Free amine
</td>
<td>
Racemic analytical standard with all diastereoisomers:
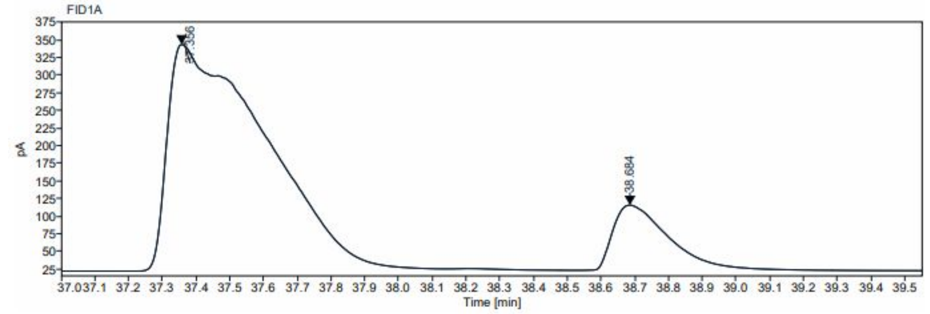

Enantiomerically enriched analytical standard:
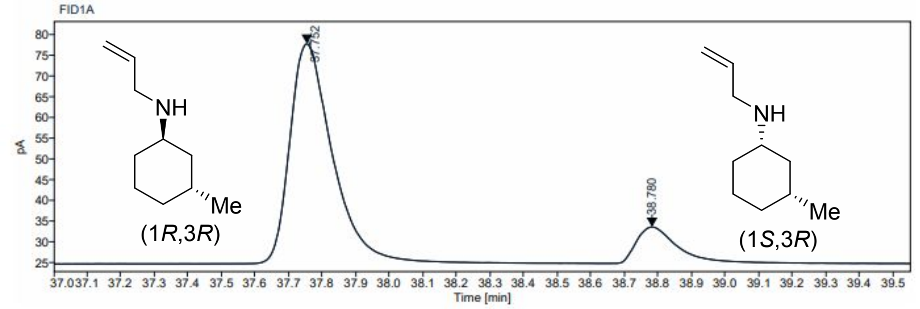

Biotransformation:
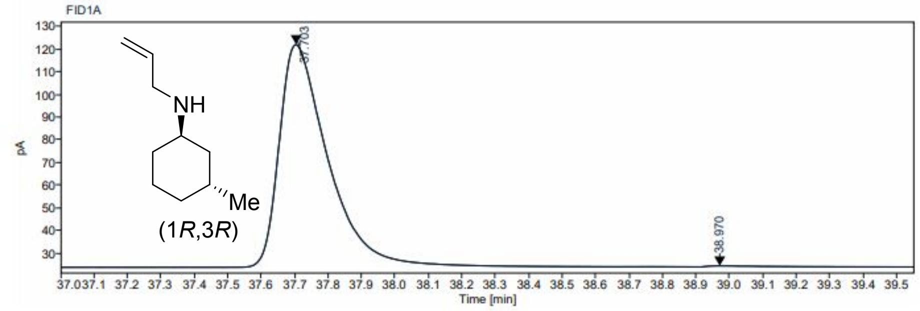

Biotransformation <u>spiked</u> with enantiomerically enriched standard:
</td>
<td>95%</td>
<td>>99:1</td>
</tr>
</table>

	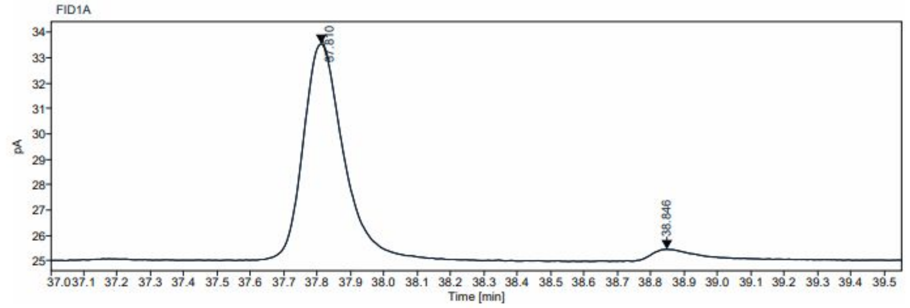FID1A
37.810
38.846
pA
Time [min]		

4.4 GCMS Time course data

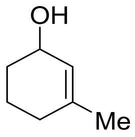

1a
RT = 3.212 min

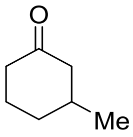

4a
RT = 3.502 min

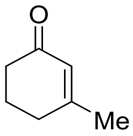

2a
RT = 3.833 min

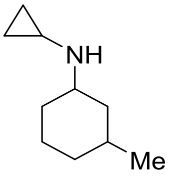

3a
RT = 4.599 min

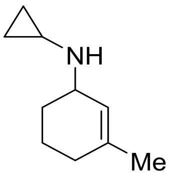

5a
RT = 5.096 min

Time point	Chromatogram
15 min	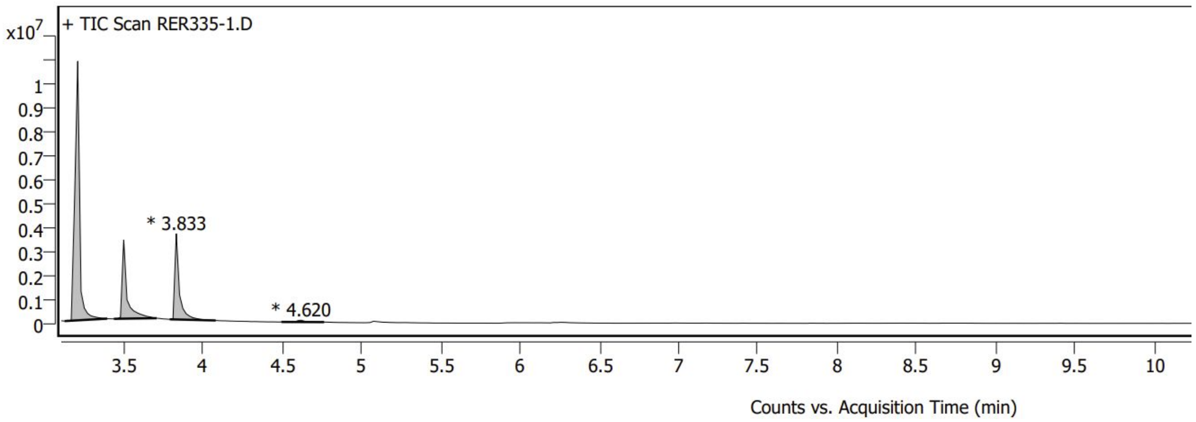
45 min	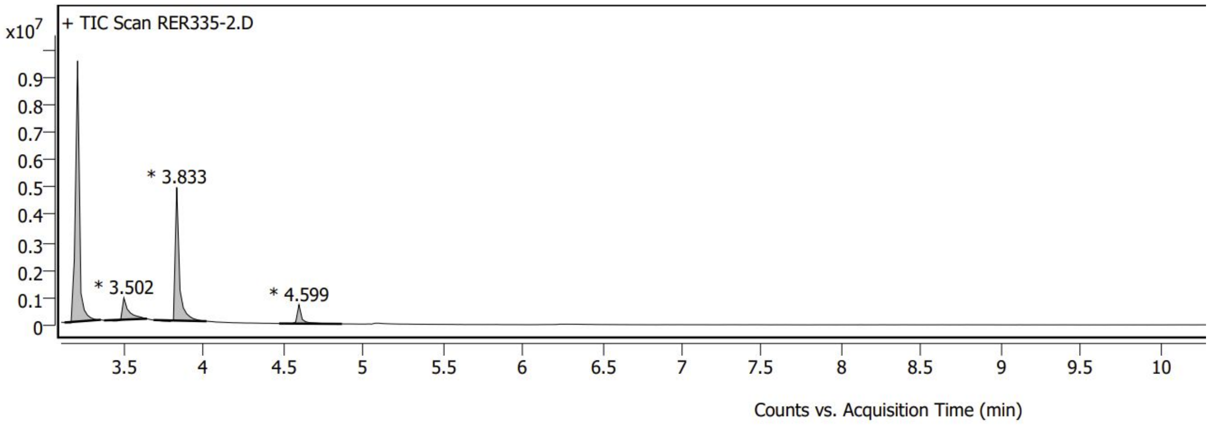
1.5 h	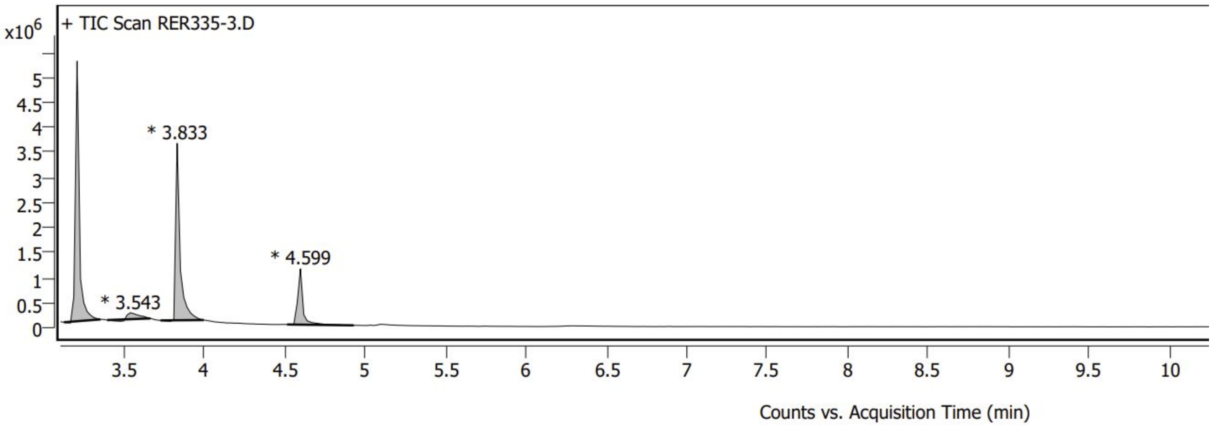

2.5 h

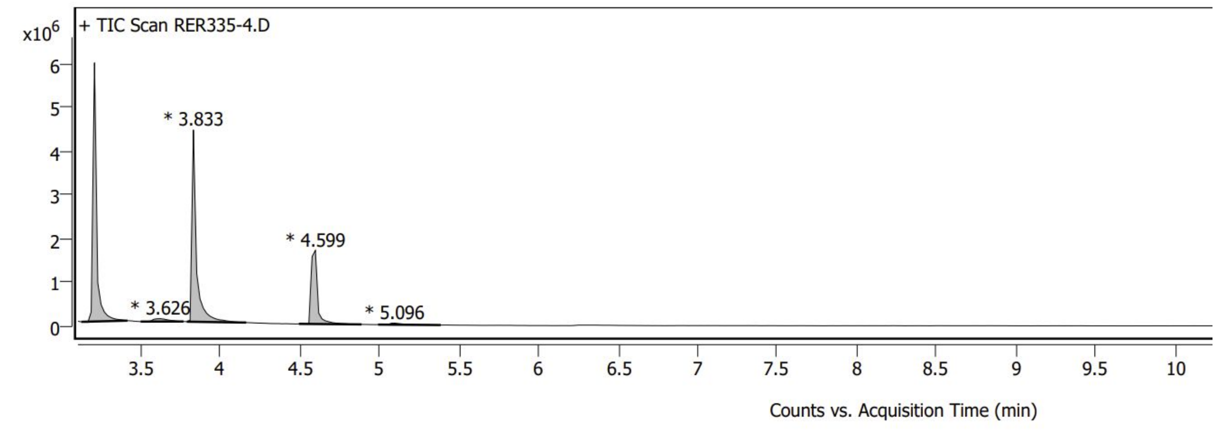

3.5 h

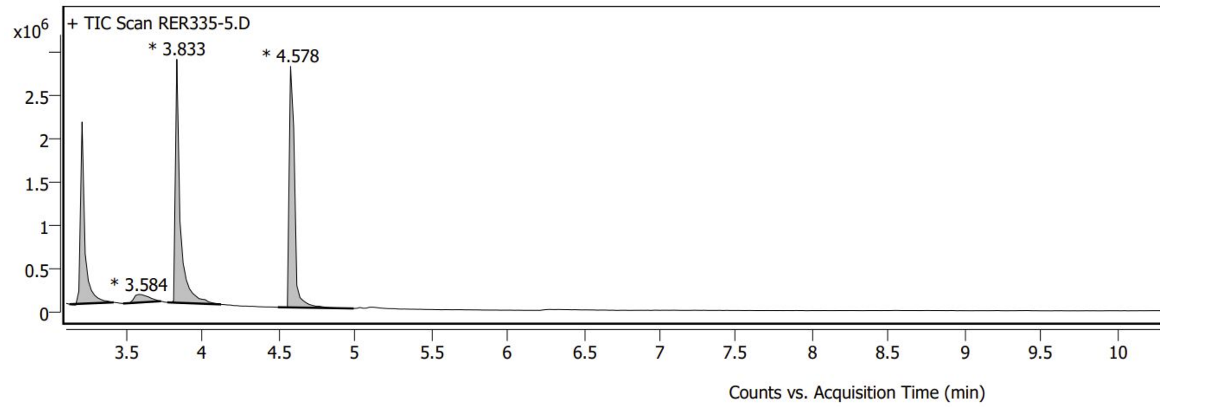

4.5 h

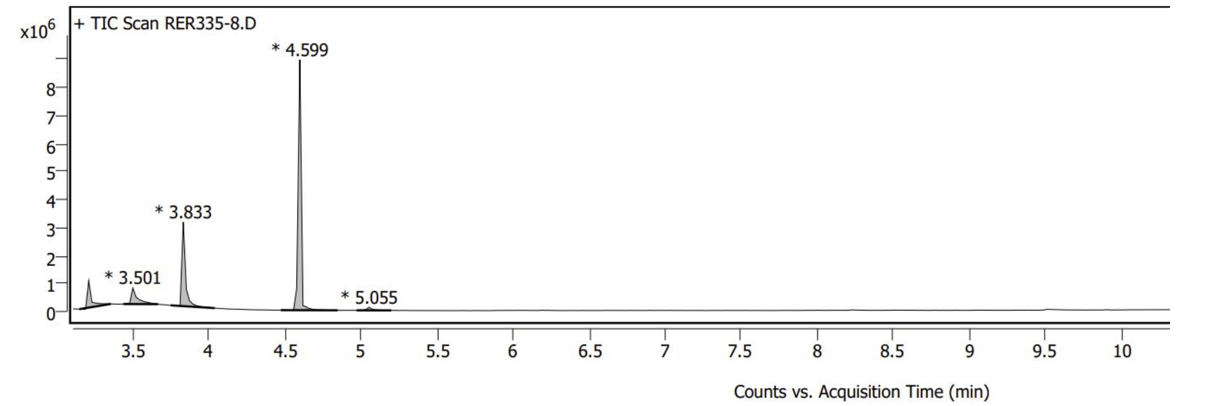

24h

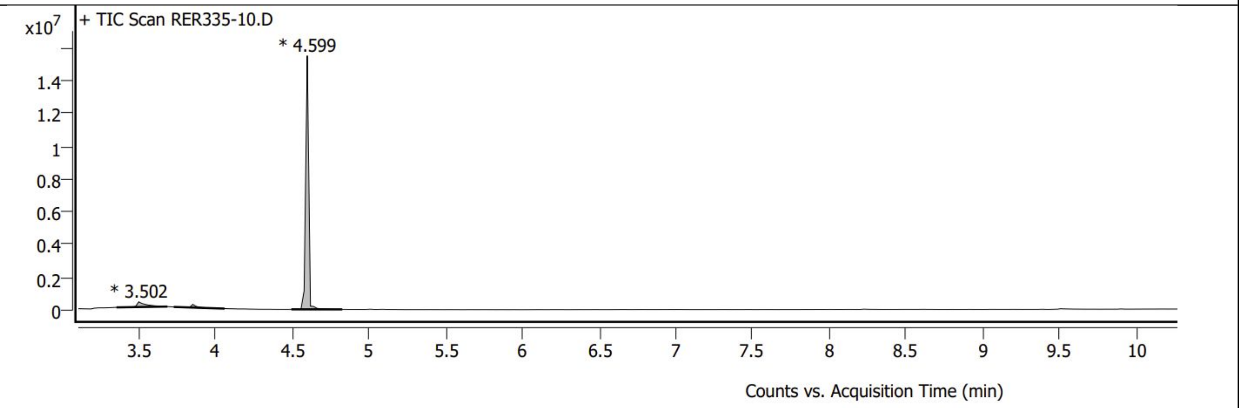

4.3 Product Distributions from GCMS Data in Table Format

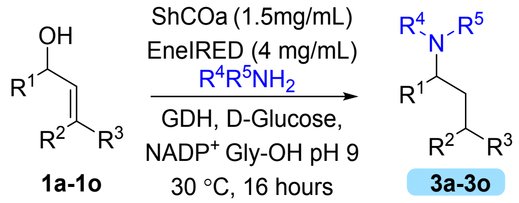

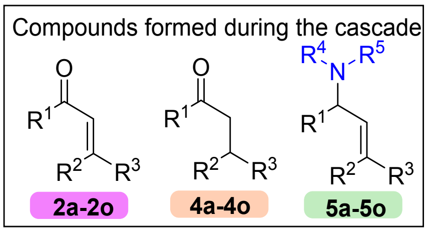

		Product Distribution (% from GCMS)						
Allylic Alcohol (1a-o)	Amine	**(1x)** Allylic alcohol	**(2x)** Enal/enone	**(3x)** Amine	**(4x)** CR intermediate	**(5x)** RA By-product	Aldimine 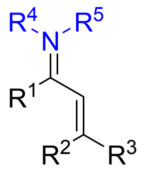	ee/dr of **3x**
**1g**	Cyclopropylamine	5		92			3	
**1a**	Cyclopropylamine	1		99				>99:1 dr >99% ee
**1b**	Cyclopropylamine	4	6	90				98:2 dr 99% ee
**1c**	Cyclopropylamine	6		94				>99:1 dr
**1d**	Cyclopropylamine	64	11	25				>99:1 dr
**1e**	Cyclopropylamine	55		25		20		83:17 dr
**1e**[a]	Cyclopropylamine	50	27	23				nd
**1f**	Cyclopropylamine	<1		>99				>99:1 dr
**1j**	Cyclopropylamine	<1		76		23		
**1a**	Allylamine	14		86				>99:1 95.5% ee
**1a**	rac-3-fluoropyrrolidine	1	3	94	2			99:<1:1:<1 dr
**1k**	Cyclopropylamine	<1		>99				
**1l**	Cyclopropylamine	<1		75		25		
**1l**	Allylamine	27		49		23		
**1o (Nerol)**	Cyclopropylamine	<1		90			10	
**1o' Geraniol)**	Cyclopropylamine	<1		3			97	

[a] reaction carried out with ShCOb

4.4 GCMS Calibration

GC-MS calibration curves were generated for the following compounds:

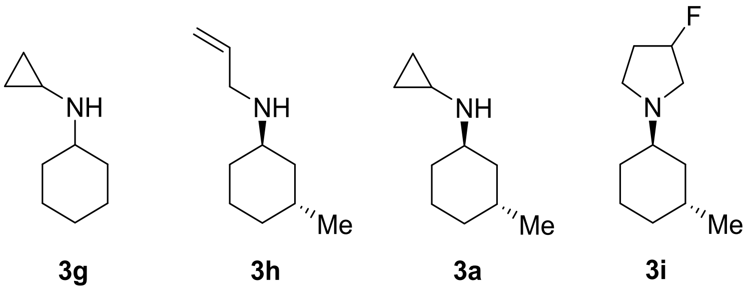

Calibrations were performed by measuring the peak areas of each compound at five concentrations of authenticated standards: 2 mM, 4 mM, 6 mM, 8 mM, and 10 mM. Each concentration was analysed in triplicate, and solvent blanks were included to establish the zero point.

One example of the full curve and data is shown below. All other examples were performed using the same data treatment.

Data for 3h shown in full:

Concentration (mM)	Area of samples run in triplicate		
10	11892270.5	12916415.52	12919176.67
8	11537297.37	10707326.76	10856160.52
6	7894119.83	7984117.84	8217504.74
4	5740725.67	5388876.06	5586647.09
2	3328690.42	2857380.3	3321374.19
0	657.09	620.46	654.23

The concentrations were plotted against the area (see below) with a line of best fit with the following equation

$$y = 1.2693x + 0.3837$$

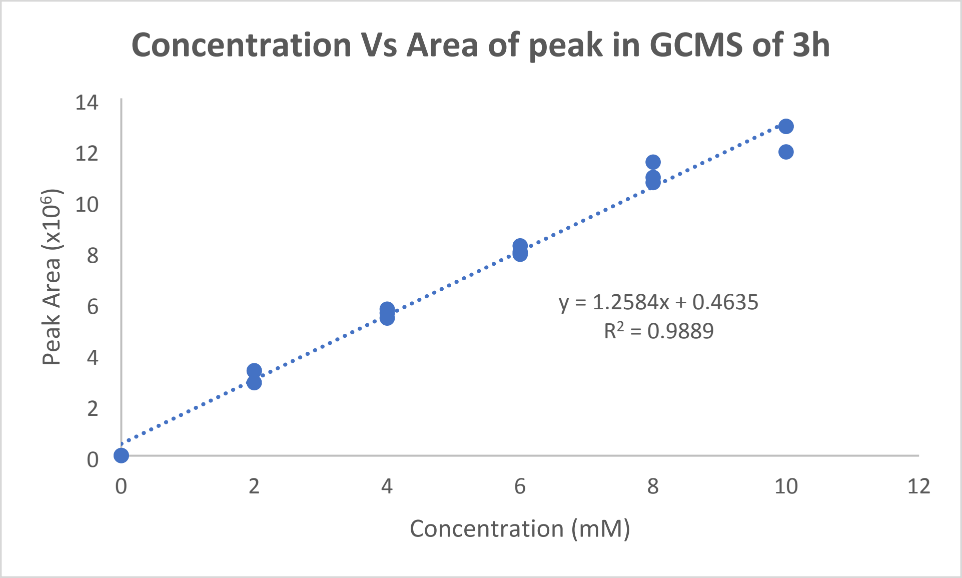

Area of peak from GCMS of biotransformation = 8420392.33 = 8.42 x 10^6

$$\boldsymbol{x = 8.0363 \div 1.2693 = 6.33\ mM}$$

Therefore, GCMS yield = 63%

Data treatment for all other compounds was performed in the same manner to give the following curves:

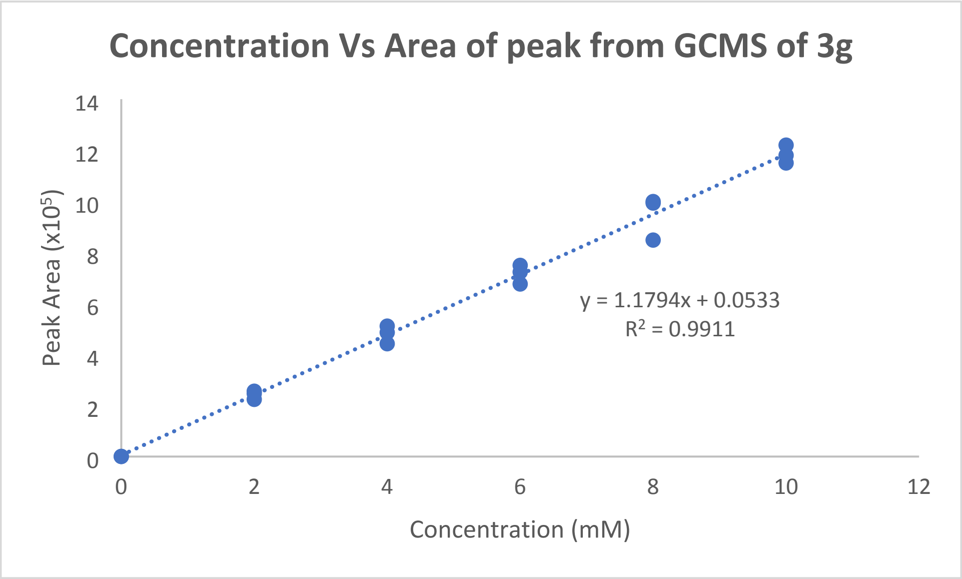
Concentration Vs Area of peak from GCMS of 3g
Peak Area (x10^5)
Concentration (mM)
y = 1.1794x + 0.0533
R² = 0.9911
0
2
4
6
8
10
12
14

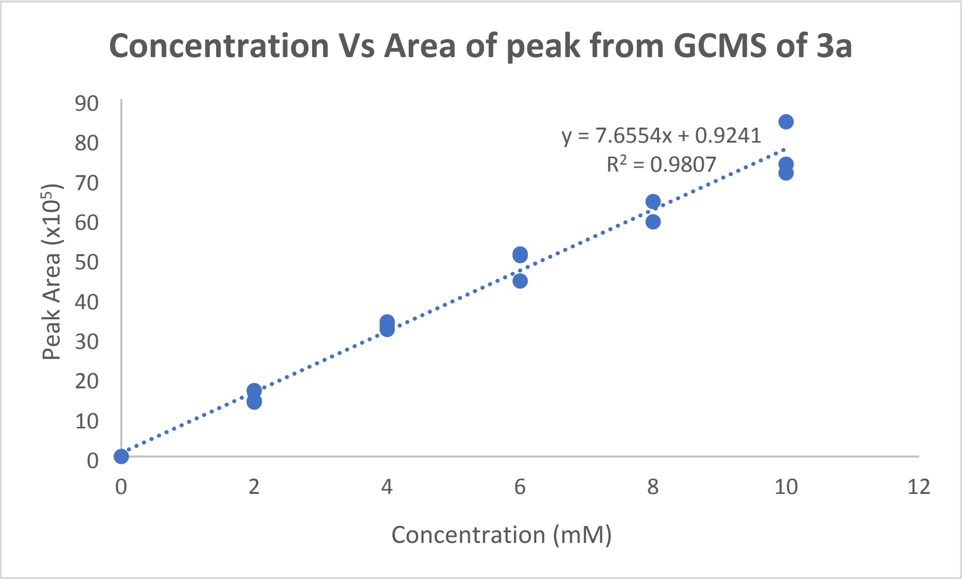
Concentration Vs Area of peak from GCMS of 3a
Peak Area (x10^5)
Concentration (mM)
y = 7.6554x + 0.9241
R² = 0.9807
0
10
20
30
40
50
60
70
80
90

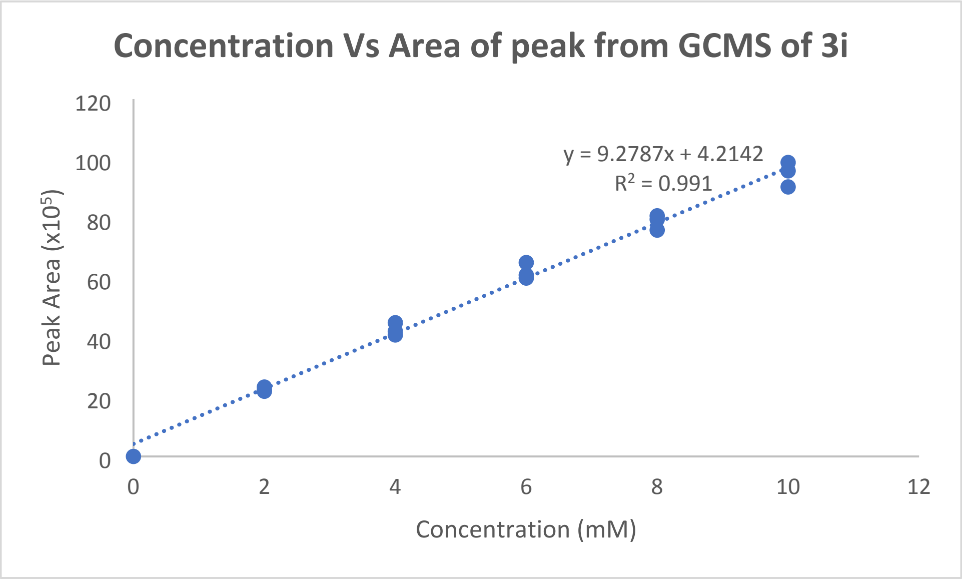
Concentration Vs Area of peak from GCMS of 3i
Peak Area (x10^5)
Concentration (mM)
y = 9.2787x + 4.2142
R² = 0.991
0
20
40
60
80
100
120

**Compound**	**Equation**	**Calc. concentration (mM)**	**Uncalibrated conversion**	**GCMS calibrated yield**
**3h**	$y = 1.2693x + 0.3837$	6.33	86%	63%
**3g**	$y = 1.1794x + 0.0533$	8.24	92%	82%
**3a**	$y = 7.6554x + 0.9241$	9.92	>99%	99%
**3i**	$y = 9.2787x + 4.2142$	9.00	94%	90%

5.0 References

(1) Heath, R. S.; Sangster, J. J.; Turner, N. J. *ChemBioChem* **2022**, *23*, e202200075.

(2) Thorpe, T.; Marshall, J.; Harawa, V.; Ruscoe, R.; Cuetos, A.; Finnigan, J.; Angelastro, A.; Parmeggiani, F.; Charnock, S.; Howard, R.; Kumar, R.; Daniels, D.; Grogan, G.; Heath, R. *Nature* **2022**, *604*, 86.

(3) Topp, C.; Metzler, J. M.; Dressler, F.; Niedek, D.; Schuler, S. M. M.; Schreiner, P. R. *Org Lett* **2024**, *26* (3), 577–580.

(4) Tsuchimochi Shuhei; Takeuchi Yasuo; Egi Masahiro; Satoh Tomo-o; Kanomata Kyohei; Ikawa Takashi; Akai Shuji, I. H. *Synlett* **2021**, *32* (08), 822–828.

(5) Wagner, B.; Binder, F. P. C.; Jiang, X.; Mühlethaler, T.; Preston, R. C.; Rabbani, S.; Smieško, M.; Schwardt, O.; Ernst, B. *Molecules* **2023**, *28* (6).

(6) Wang, C.; Dong, G. *J Am Chem Soc* **2018**, *140* (19), 6057–6061.

(7) Song, L.; Zhou, Y.; Liang, H.; Li, H.; Lai, Y.; Yao, H.; Lin, R.; Tong, R. *J Org Chem* **2023**, *88* (1), 504–512.

(8) Choi, S.; Narayanasamy, P. *Bioorg Med Chem Lett* **2021**, *47*, 128203.

(9) Montgomery, S. L.; Mangas-Sanchez, J.; Thompson, M. P.; Aleku, G. A.; Dominguez, B.; Turner, N. J. *Angewandte Chemie International Edition* **2017**, *56* (35), 10491–10494.

(10) Ramsden, J. I.; Heath, R. S.; Derrington, S. R.; Montgomery, S. L.; Mangas-Sanchez, J.; Mulholland, K. R.; Turner, N. J. *J Am Chem Soc* **2019**, *141* (3), 1201–1206.

6.0 NMR Spectra of New Compounds

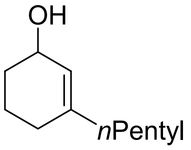

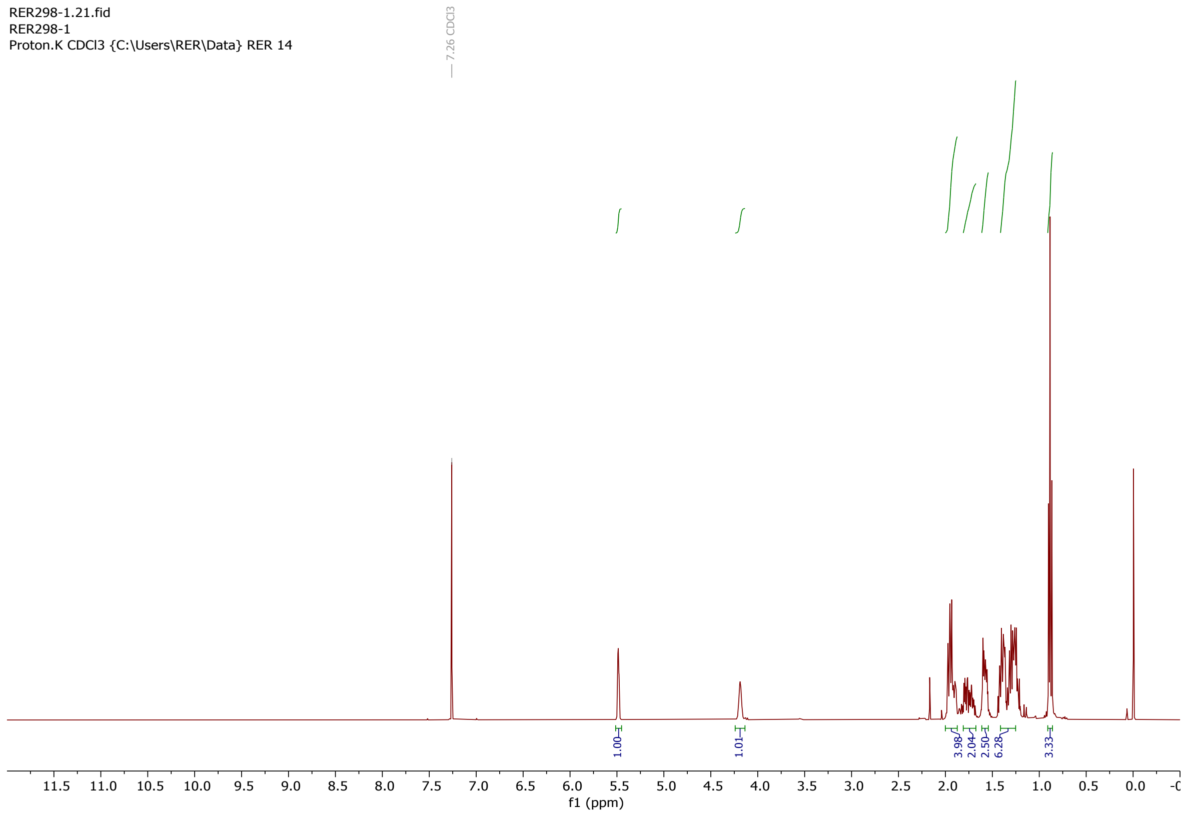

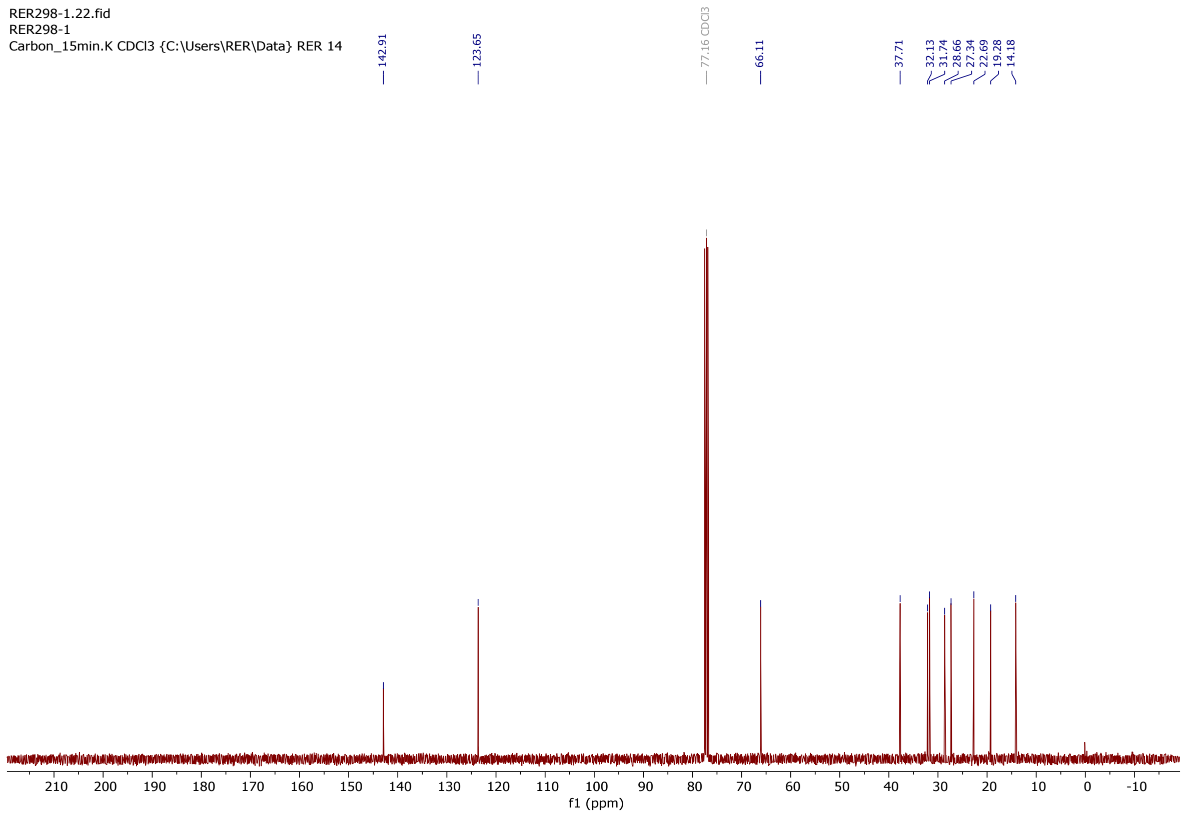

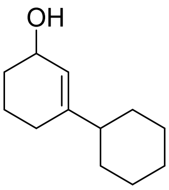
OH

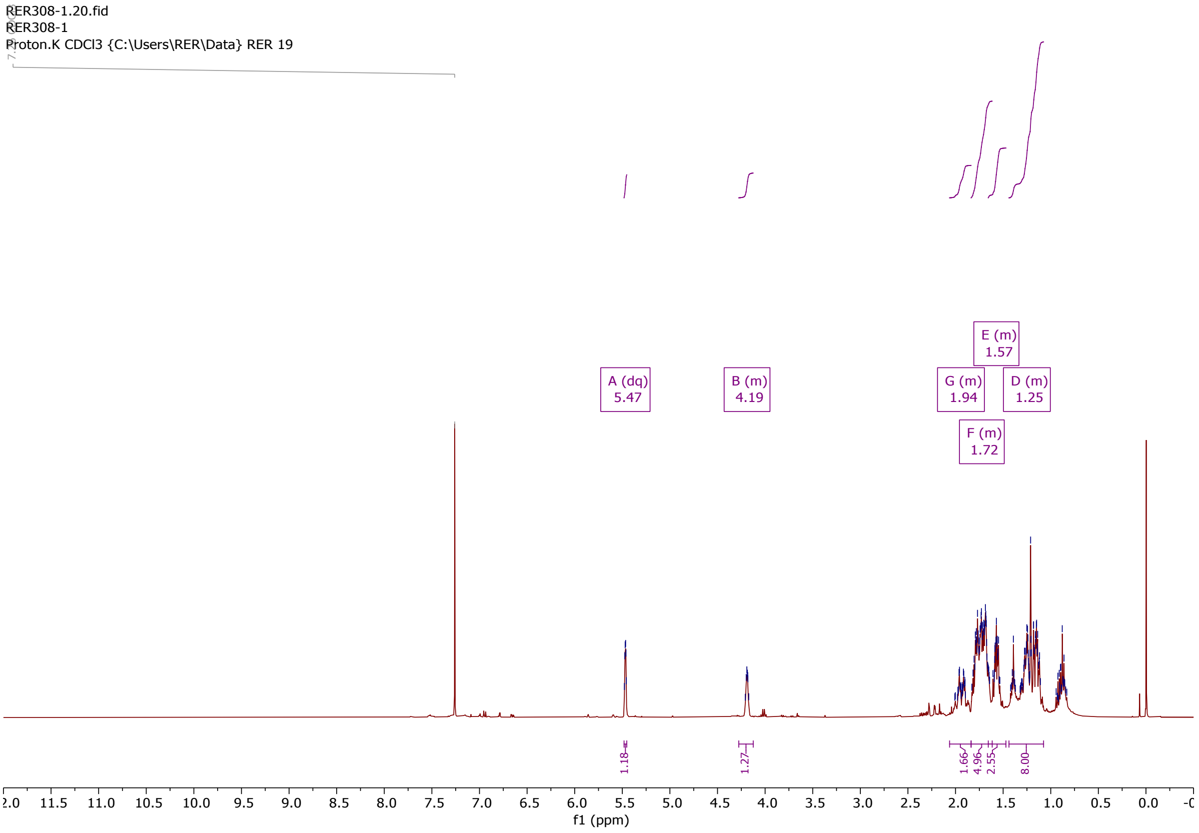
RER308-1.20.fid
RER308-1
Proton.K CDCl3 {C:\Users\RER\Data} RER 19
A (dq)
5.47
B (m)
4.19
E (m)
1.57
G (m)
1.94
D (m)
1.25
F (m)
1.72
1.18
1.27
1.66
4.96
2.55
8.00
f1 (ppm)

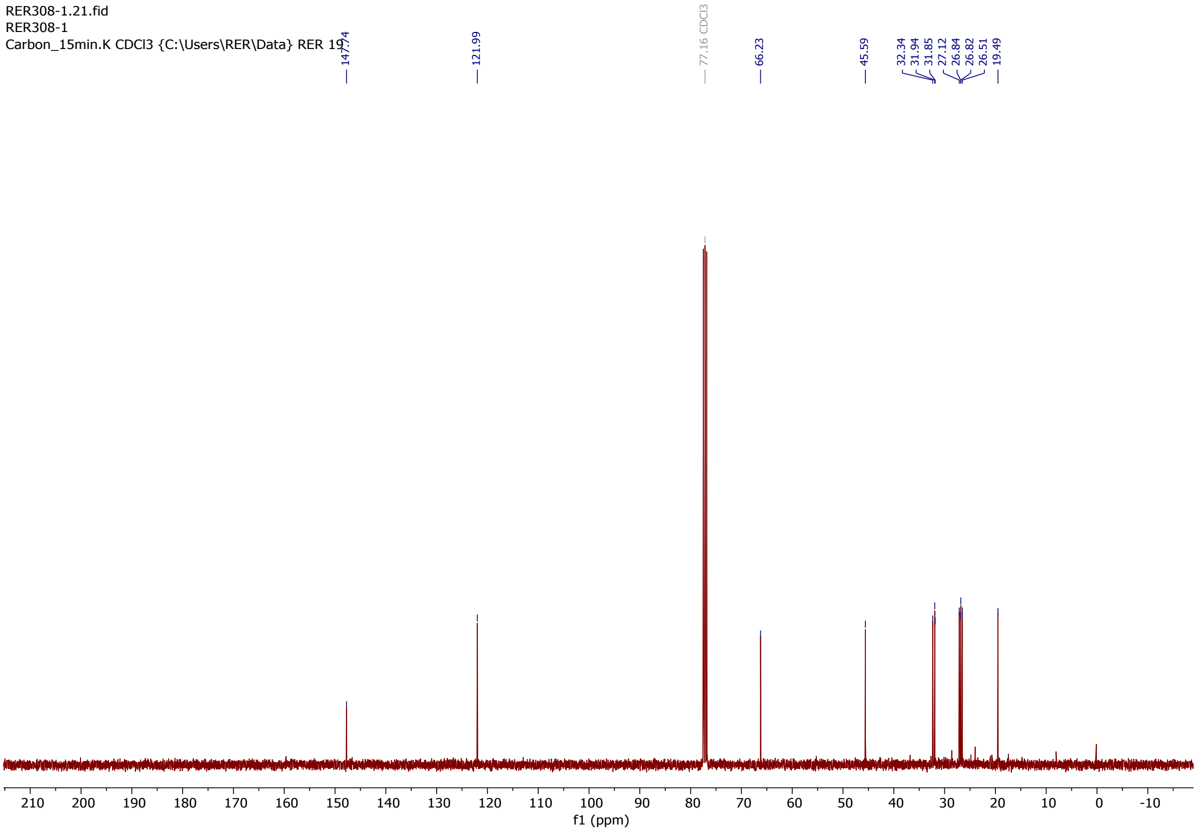
RER308-1.21.fid
RER308-1
Carbon_15min.K CDCl3 {C:\Users\RER\Data} RER 19
147.74
121.99
77.16 CDCl3
66.23
45.59
32.34
31.94
31.85
27.12
26.84
26.82
26.51
19.49
f1 (ppm)
